# Transcriptomic and metabolomic analyses reveal that ABA increases the salt tolerance of rice significantly correlated with jasmonic acid biosynthesis and flavonoid biosynthesis

**DOI:** 10.1038/s41598-023-47657-w

**Published:** 2023-11-21

**Authors:** Chunning Han, Guanjie Chen, Dianfeng Zheng, Naijie Feng

**Affiliations:** 1 College of Coastal Agricultural Sciences, Guangdong Ocean University, Zhanjiang, 524088 China; 2 Shenzhen Research Institute of Guangdong Ocean University, Shenzhen, 518108 China; 3 South China Center of National Salt-Alkali Tolerant Rice Technology Innovation Center, Zhanjiang, 524088 Guangdong China; 4 School of Tropical Crops, Hainan University, Haikou, 570228 China

**Keywords:** Molecular biology, Physiology

## Abstract

Abscisic acid (ABA) has been shown to mitigate the deleterious effects of abiotic stresses and to regulate plant growth and development. Salinity is one of the important abiotic stresses affecting plant cell metabolism and physiology, which causes serious damages to crops. In this study, we investigated the protective role of exogenous ABA on leaves in response to salinity stress using rice seedlings (two leaf-one heart) subjected to three treatments: ZCK (control), ZS (50 mM NaCl), and ZSA (5 mg L^–1^ ABA + 50 mM NaCl). We carried out transcriptomic and metabolomic analyses to identify the molecular mechanisms by which ABA protects plants against salt stress. Results showed that 1159 differentially expressed genes (DEGs) (916 up-regulated, 243 down-regulated) and 63 differentially accumulated metabolites (DAMs) (42 up-regulated, 21 down-regulated) were identified between the ZS and ZSA treatments, respectively. In addition, ABA pretreatment regulated the expression pattern of genes responsible for oxidation redox, starch and sucrose metabolism, and phenylpropanoid biosynthesis. The combined transcriptomic and metabolomic analysis revealed that 16 DEGs and 2 DAMs were involved in Flavonoid biosynthesis and 8 DEGs and 2 DAMs were involved alpha-Linolenic acid metabolism which are responsible for salinity stress tolerance through induced by exogenous ABA. Overall, ABA could enhance rice leaves growth and development mainly by regulating flavonoid biosynthesis and linoleic acid metabolism pathway.

## Introduction

Rice (*Oryza sativa* L.) is the most important staple food crops for more than half of the world population. As a salt-sensitive plant, the growth and production of rice is decreasing due to salinity. In fact, salt stress is the most important stress factor among abiotic stresses that not only leads to an imbalance in the Earth’s climatic conditions, but also has destructive effects on plant growth and physiological processes. Salinity in the soil adversely affects root growth and reduces the ability of plants to absorb water and other nutrients from the soil, thus causing delayed plants growth even ultimately lead to plants death. Actually, salt stress leads to membrane damage and stomatal closure, which results in reduced carbon dioxide uptake, hydrolase activity, and increased lipid peroxidation levels, which may stimulate a large number of formation of ROS. Admittedly, antioxidant defense system (mainly includes SOD, POD, CAT, APX) could be effectively eliminated ROS and maintain appropriate equilibrium within the cells 9, which are contributed to improve salt tolerance.

As important small molecules in regulating metabolism, hormones play a crucial role in the response to abiotic stresses^1^. Abscisic acid (ABA) is a vital endogenous plant hormone which is usually involved in stimulating various aspects of plant growth and development, such as seed dormancy and germination, root structure, leaf senescence, and vegetative growth^2^. ABA reduces stress and is essential in salt-stressed conditions due to its significant impact on the phenotypic, metabolomic, and transcriptomic responses of plants as well as high efficiency of genes responsible for flavonoid metabolism^3^. We previous study have demonstrated that exogenous ABA to rice was beneficial to protect membrane lipid peroxidation, the modulation of antioxidant defense systems and endogenous hormonal balance with imposition to salt stress^4^. In addition to a series of physiological events, many studies also focus on its molecular mechanism at transcriptomic and metabolomics level. Transcriptome analysis showed that ABA control the primary root growth in response to salt stress via inducing the expression of *EXPANSIN* genes, further investigations indicated that ABA exerts these effects largely through ABA signaling^5^. Previous study showed that exogenous ABA highly up-regulated the genes involved in the lipid and fatty acid metabolisms and cytoplasmic transport in shoots of rice under salt stress, suggesting that enhanced lipid metabolism might also contribute to enhanced salt tolerance of rice^6^. Actually, a plenty of genes that confer salt tolerance were reported in recent decades. However, the levels of salt tolerance differ greatly depending on growth conditions, and mechanisms underlying the complicated nature of stress tolerance are far from being fully understood. Additionally, an intrinsic association between genes and metabolites may also exist following salt stress injury.

The inbred rice variety "*Huanghuazhan*" is an elite indica rice cultivar, which has been grown over 4.5 million ha in southern China, it is a semi-dwarf, super high yield, good eating quality and highly adapt to vast areas of nine provinces in China, and twenty cultivars have been derived from it^7^. The present study investigated that exogenous ABA on leaves (foliar spraying) in response to salt stress using rice variety ‘*Huanghuazhan*’ (two leaf-one heart). We carried out transcriptomic and metabolomic analyses to identify the molecular mechanisms by which ABA protects plants against salt stress. By using omics technology, we identified specific genes, secondary metabolites that are targeted for flavonoids and alpha-Linolenic acid. In this regards, we have obtained valuable information for understanding the salt tolerance mechanisms of rice and for the effective control of salinity stress.

## Results

### Effects of ABA on morphological parameters of rice seedlings under salt stress

**Figure 1:**
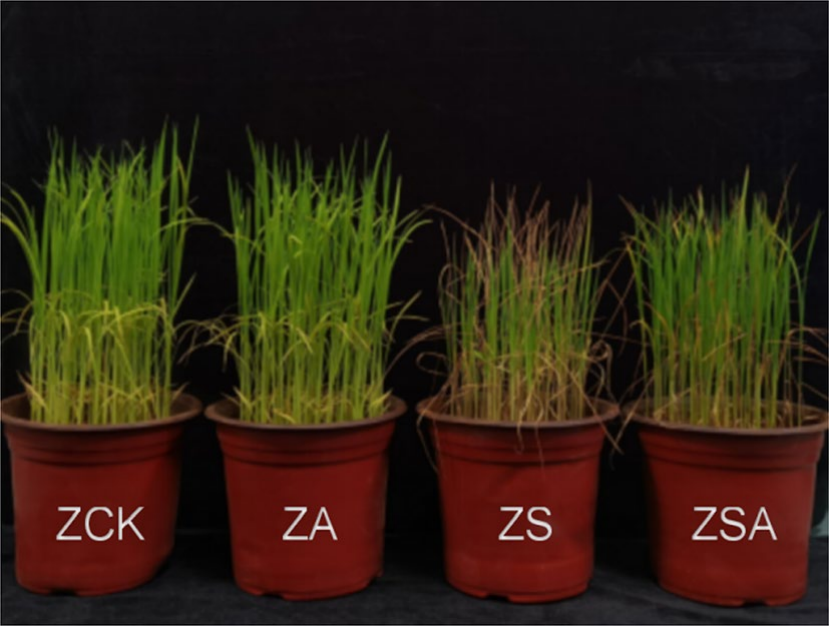
Effects of ABA on morphology of rice seedlings after 10 d of salt stress. ZCK treatment of water, ZA treatment with ABA lonely, ZS treatment with NaCl, ZSA treatment with ABA + NaCl in *Huanghuazhan*. The current study mainly investigate ZCK, ZS and ZSA.

Salt stress significantly decreased rice plant height, stem diameter, leaf length and leaf area when compared with the control. The foliar ABA application significantly increased its stem diameter, leaf length and leaf area (Table 1). Figure 1 shows the phenotype of rice seedlings at the 10th d after salt stress.

**Table 1:** Effects of ABA on shoot morphological parameters of rice seedlings under salt stress.

Treatment	Plant height (cm)	Stem diameter (mm)	Leaf length (mm)	Leaf area (mm^2^)
ZCK	31.08 ± 0.38a	3.43 ± 0.10a	144.25 ± 7.97a	180.63 ± 11.94b
ZS	27.48 ± 0.38b	2.95 ± 0.09b	101.38 ± 5.63b	118.42 ± 12.96c
ZSA	28.18 ± 0.69b	3.29 ± 0.08a	145.30 ± 7.31a	347.08 ± 26.29a

ZCK treatment of water, ZS treatment with NaCl, ZSA treatment with ABA + NaCl in *Huanghuazhan*. Data are mean ± standard error of at least four replicates. Within each column, different letters indicate significant difference at the five percent significant level according to Duncan’s multiple range tests.

Salt stress significantly decreased root length, root surface area, root volume and root average diameter in rice when compared with the control. Foliar spraying of ABA greatly increased these root morphology indicators (Table 2).

**Table 2:** Effects of ABA on root morphological parameters of rice seedlings under salt stress.

Treatment	Root length (cm)	Root surface area (cm^2^)	Root volume (cm^3^)	Root average diameter (mm^2^)
ZCK	1923.892 ± 64.187b	209.023 ± 6.303b	3.359 ± 0.122b	0.321 ± 0.004a
ZS	1345.150 ± 50.764c	136.310 ± 4.974c	1.986 ± 0.111c	0.294 ± 0.004b
ZSA	2230.447 ± 74.359a	244.669 ± 9.127a	4.156 ± 0.116a	0.328 ± 0.003a

ZCK treatment of water, ZS treatment with NaCl, ZSA treatment with ABA + NaCl in *Huanghuazhan*. Data are mean ± standard error of at least four replicates. Within each column, different letters indicate significant difference at the five percent significant level according to Duncan’s multiple range tests.

### Effects of ABA on rice seedling biomass under salt stress

**Figure 2:**
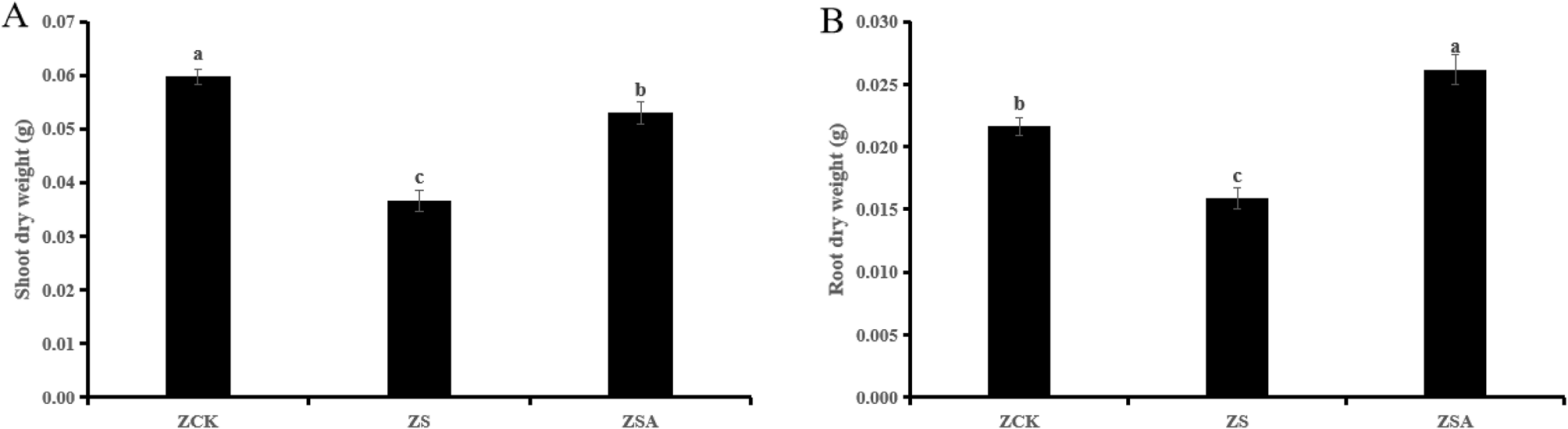
Effects of ABA on shoot dry weight (**A**) and root dry weight (**B**) of rice seedlings under salt stress. Different letters indicate significant difference at the five percent significant level according to Duncan’s multiple range tests.

Compared with CK, salt stress significantly reduced the shoot dry weight and root dry weight of rice. ABA + NaCl significantly increased its shoot dry weight and root dry weight compared with the salt treatment alone (Fig. 2).

### DEGs and DAMs induced by ABA pretreatment in response to salt stress

**Figure 3:**
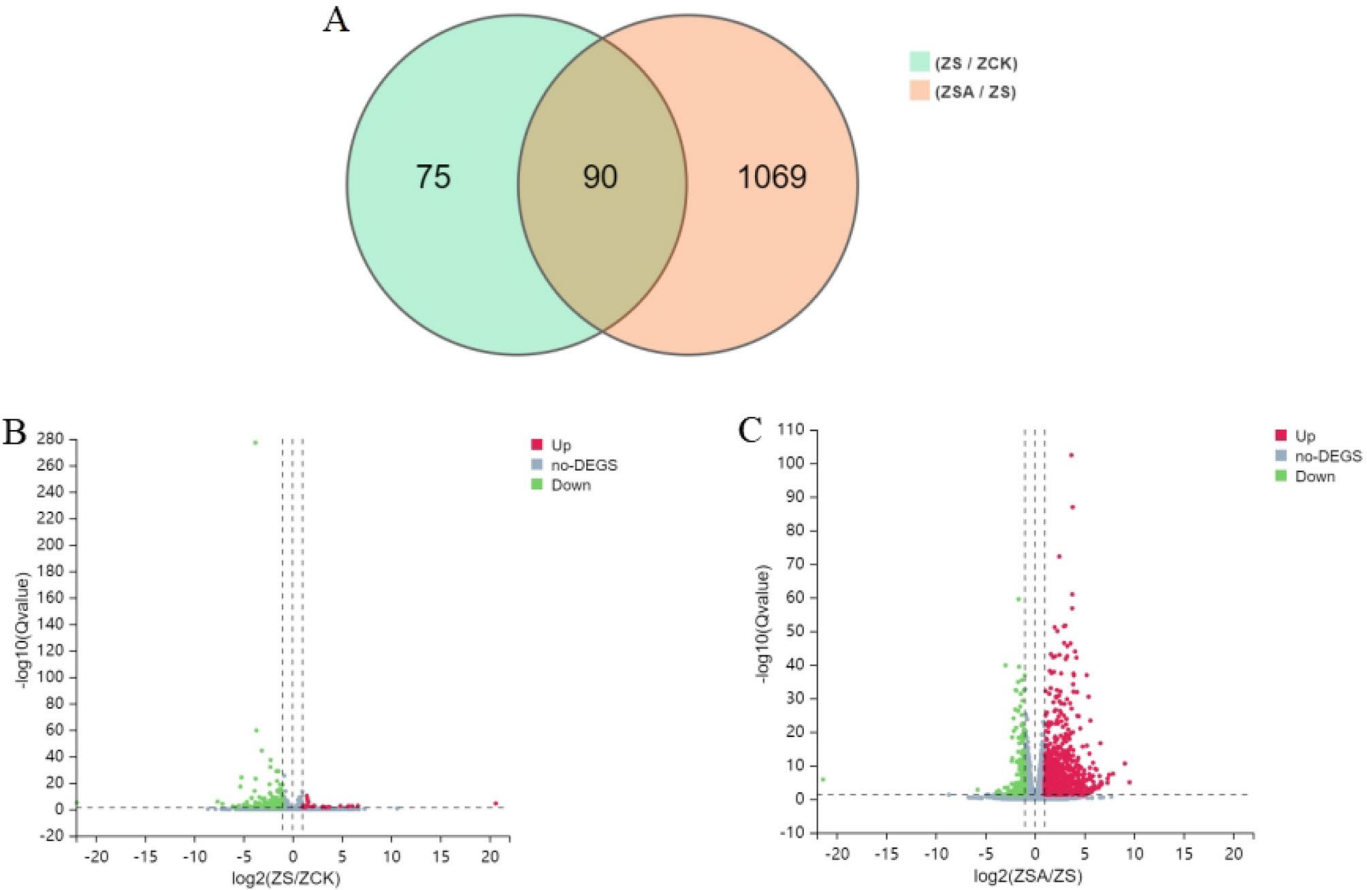
Venn diagram (**A**) and the volcano maps (**B**,**C**) of DEGs under salt stress and ABA treatment. The X axis represents the log2 transformed difference-fold value, and the Y axis represents the-log10 transformed significance value. Red represents up-regulated DEG, blue represents down-regulated DEG, and gray represents non-DEG.

To investigate the effects of ABA treatment on gene transcription under salt stress, mRNA sequencing was performed. Nine samples were sequenced through BGISEQ. An average of 95.08% clean reads were generated in each sample, corresponding to a total of 6.46G Gb clean bases. Total 83.18% to 84.37% of clean reads were mapped to reference genome (Table S2). We identified 165 DEGs (24 up and 141 down) by comparing NaCl and CK treatments, while 1159 DEGs (916 up and 243 down) were identified by comparing the ABA + NaCl and NaCl treatments (Fig. 3). These results suggested that the genes expressed to exogenous ABA would be considered important candidates gene for further analysis.

**Figure 4:**
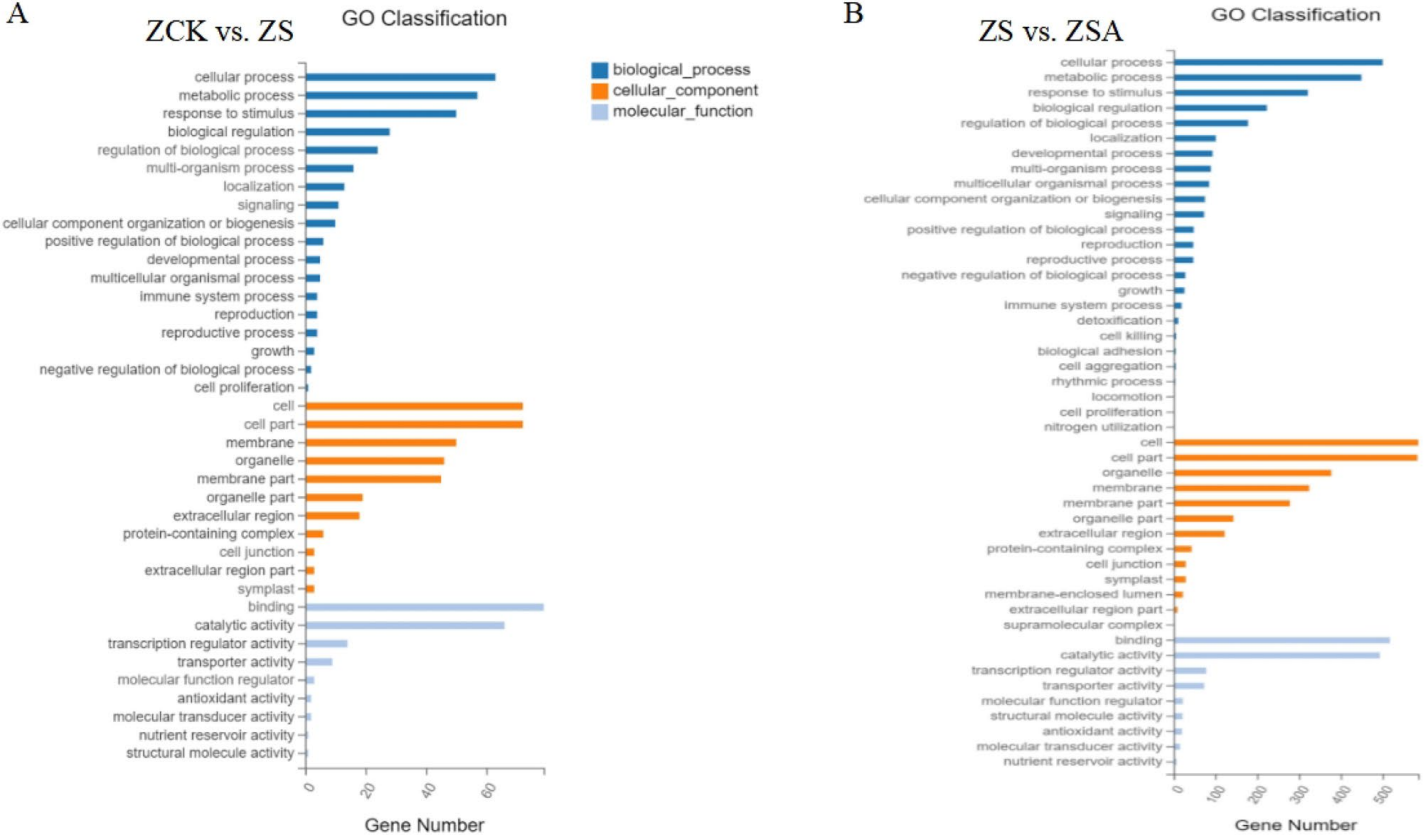
GO classification of differentially expressed genes between the control and NaCl group (**A**), NaCl and NaCl + ABA treatment group (**B**) after 72 h of foliar sprayed ABA.

GO analysis of the DEGs revealed that control, NaCl, and NaCl + ABA treatments enriched GO terms biological processes, cellular components and molecular function (Fig. 4). In the category of biological process, the DEGs of the control and salinity treatment, salinity and NaCl + ABA treatment group mainly distributed in cellular, metabolic process and response to stimulus while the DEGs in all the treatments had the highest proportion in cell and cell part in the category of cellular components. Additionally, DEGs mainly distributed in binding and catalytic activity in the category of molecular function (Fig. 4).

GO annotations were used to classify the possible functions of the rice genes. Enriched analysis were performed to obtain Q-value (≤ 0.05 significant) through the phy.per function in the R software. The GO enrichment analysis of 165 and 1159 DEGs in ZCK vs. ZS and ZS vs. ZSA, respectively, classified GO into three categories such as biological process (BP), cellular component (CC), and molecular function (MF). GO analysis of the DEGs indicated significant enriched GO terms in BP, CC and MF (Table 3). GO terms in the MF and BP were significantly enriched in ZCK vs. ZS and ZS vs. ZSA. Similarly, compared to ZCK vs. ZS, the number of enriched GO terms in the MF and BP was found higher in ZS vs. ZSA. These results indicated that the application of ABA plays an important role in regulating MF and BP gene expression under salinity stress. Additionally, combined ZCK vs. ZS and ZS vs. ZSA, the number of GO terms in the BP category were found higher among the three categories. It is obvious that the biological process related to defense response, catabolic process and carbohydrate metabolism were most influenced by salinity stress.

**Table 3:** The significantly enriched GO pathways.

Combination	Category	GO ID	Description	Q-value
ZCK vs. ZS	MF	GO:0004568	Chitinase activity	0.007
		GO:0004072	Aspartate kinase activity	0.030
	BP	GO:0000272	Polysaccharide catabolic process	0.006
		GO:0006032	Chitin catabolic process	0.006
		GO:0006952	Defense response	0.006
		GO:0009620	Response to fungus	0.006
		GO:0010200	Response to chitin	0.006
		GO:0010438	Cellular response to sulfur starvation	0.006
		GO:0009090	Homoserine biosynthetic process	0.011
		GO:0050832	Defense response to fungus	0.011
		GO:0010439	Regulation of glucosinolate biosynthetic process	0.012
		GO:0009617	Response to bacterium	0.015
		GO:0009088	Threonine biosynthetic process	0.022
		GO:0009751	Response to salicylic acid	0.025
		GO:0009814	Defense response, incompatible interaction	0.030
		GO:0009089	Lysine biosynthetic process via diaminopimelate	0.036
		GO:0010104	Regulation of ethylene-activated signaling pathway	0.042
		GO:0034488	Basic amino acid transmembrane export from vacuole	0.048
		GO:1900706	Positive regulation of siderophore biosynthetic process	0.048
ZS vs. ZSA	CC	GO:0005576	Extracellular region	8.16E-06
		GO:0031225	Anchored component of membrane	0.050
		GO:0042764	Ascospore-type prospore	0.050
		GO:0046658	Anchored component of plasma membrane	0.050
		GO:0048046	Apoplast	0.050
	MF	GO:0003700	DNA-binding transcription factor activity	1.82E-04
		GO:0004568	Chitinase activity	5.66E-04
		GO:0004867	Serine-type endopeptidase inhibitor activity	0.002
		GO:0052716	Hydroquinone:oxygen oxidoreductase activity	0.002
		GO:0004364	Glutathione transferase activity	0.014
		GO:0015152	Glucose-6-phosphate transmembrane transporter activity	0.014
		GO:0016298	Lipase activity	0.014
		GO:0004601	Peroxidase activity	0.017
		GO:0042973	Glucan endo-1,3-beta-D-glucosidase activity	0.018
		GO:0102336	3-oxo-arachidoyl-CoA synthase activity	0.018
		GO:0102337	3-oxo-cerotoyl-CoA synthase activity	0.018
		GO:0102338	3-oxo-lignoceronyl-CoA synthase activity	0.018
		GO:0102756	Very-long-chain 3-ketoacyl-coa synthase activity	0.018
		GO:0043565	Sequence-specific dna binding	0.026
		GO:0009055	Electron transfer activity	0.030
		GO:0030170	Pyridoxal phosphate binding	0.035
		GO:0015120	Phosphoglycerate transmembrane transporter activity	0.044
		GO:0071917	Triose-phosphate transmembrane transporter activity	0.044
		GO:0020037	Heme binding	0.045
		GO:0004322	Ferroxidase activity	0.045
	BP	GO:0010200	Response to chitin	8.59E-05
		GO:0009643	Photosynthetic acclimation	0.007
		GO:0031640	Killing of cells of other organism	0.007
		GO:0050832	Defense response to fungus	0.007
		GO:2000022	Regulation of jasmonic acid mediated signaling pathway	0.007
		GO:0046274	Lignin catabolic process	0.008
		GO:0000272	Polysaccharide catabolic process	0.008
		GO:0006979	Response to oxidative stress	0.008
		GO:0006032	Chitin catabolic process	0.010
		GO:0042744	Hydrogen peroxide catabolic process	0.010
		GO:0071456	Cellular response to hypoxia	0.010
		GO:0006749	Glutathione metabolic process	0.010
		GO:0009416	Response to light stimulus	0.010
		GO:0015713	Phosphoglycerate transmembrane transport	0.010
		GO:0015760	Glucose-6-phosphate transport	0.010
		GO:0009695	Jasmonic acid biosynthetic process	0.010
		GO:0006561	Proline biosynthetic process	0.016
		GO:0006587	Serotonin biosynthetic process from tryptophan	0.016
		GO:0006805	Xenobiotic metabolic process	0.016
		GO:0010438	Cellular response to sulfur starvation	0.016

**Figure 5:**
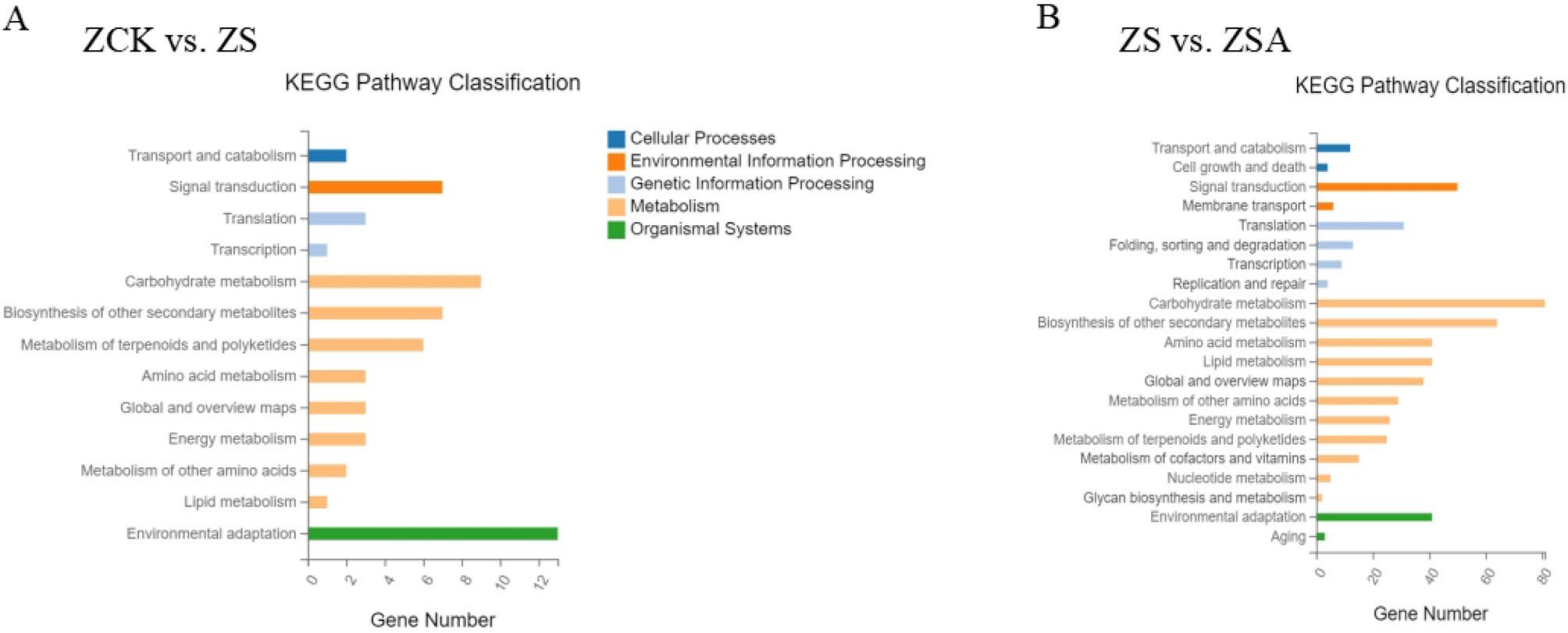
KEGG classification of differentially expressed genes between the control and NaCl group (**A**), NaCl and NaCl + ABA treatment group (**B**) after 72 h of foliar sprayed ABA.

Furthermore, we performed a KEGG pathway analysis for DEGs. The 165 DEGs (ZCK vs. ZS) with KEGG annotation were assigned to 18 pathways and 1159 DEGs (ZS vs. ZSA) were assigned to 21 KEGG pathways. The annotation results were classified by pathway type and it was observed that the pathways of DEGs were basically related to metabolism (Fig. 5). Comparative analysis of ZCK vs. ZS and ZS vs. ZSA, showed that DEGs were significantly differ in environmental adaptation, carbon metabolism processes and biosynthesis of other secondary metabolites. We explored key genes differentially expressed in rice leaves after salt stress and ABA treatment from these metabolic pathways.

**Figure 6:**
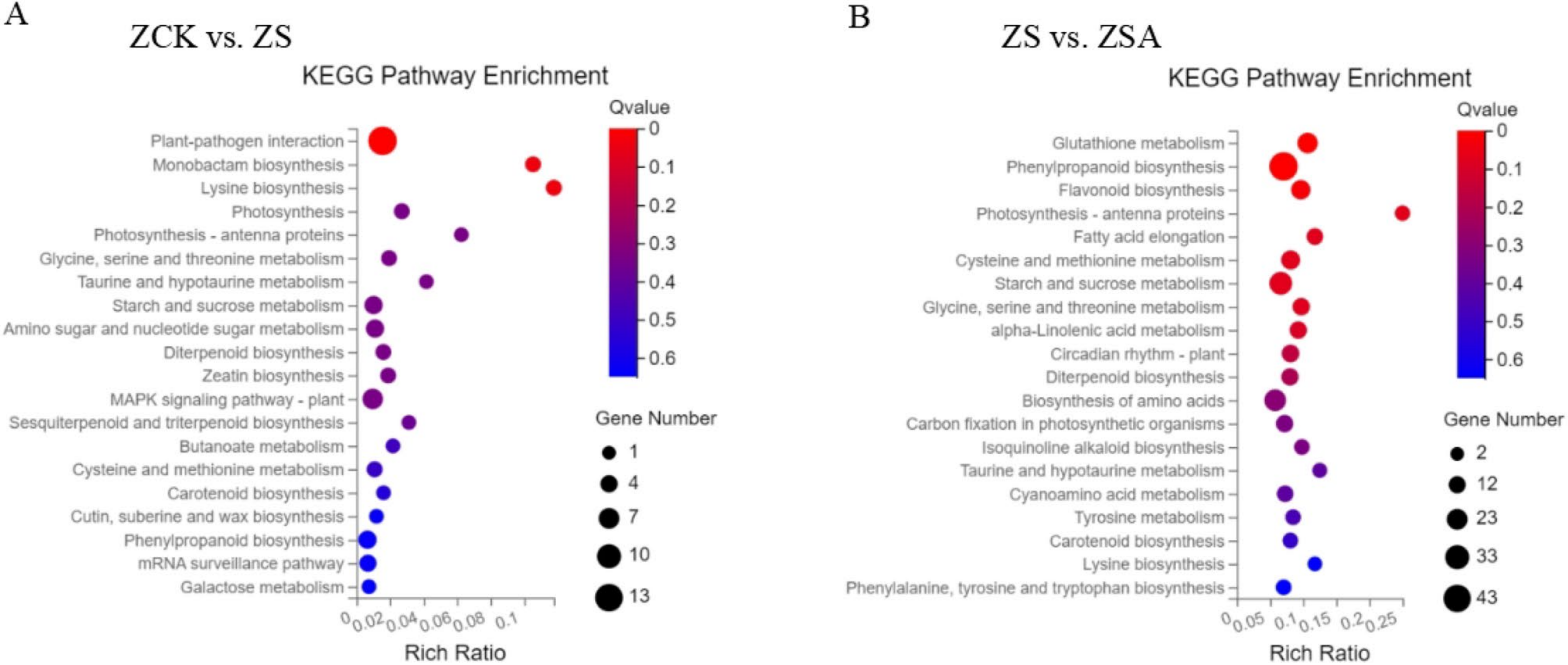
Functional analysis of DEGs based on KEGG pathway annotations. Pathways with a Q-value ≤ 0.05 that were significantly enriched in DEGs between the control and NaCl group (**A**), NaCl and NaCl + ABA treatment group (**B**) after 72 h of foliar sprayed ABA, were analyzed with the KEGG database.

For the KEGG enrichment analysis, pathways with a Q value ≤ 0.05 were regarded as significantly changed in response to salinity stress with or without ABA treatment. The top 20 enriched KEGG pathways are presented in Fig. 6. In ZCK vs. ZS, the pathways among the DEGs including plant-pathogen interaction (Os04626), with 13 genes down-regulated in ZS (Supplementary Table S3), Monobactam biosynthesis (Os00261), with 2 genes down-regulated in ZS, and Lysine biosynthesis (Os00300), with 2 genes down-regulated in ZS (Supplementary Table S4), were the major enriched pathways among the DEGs. In ZS vs. ZSA, including Glutathione metabolism (Os00480), with 19 genes (17 genes up-regulated and 2 genes down-regulated in ZSA) (Supplementary Table S5), phenylpropanoid biosynthesis (Os00940), with 43 genes (39 genes up-regulated and 4 genes down-regulated in ZSA) (Supplementary Table S6), Flavonoid biosynthesis (Os00941), with 16 genes (with 13 genes up-regulated and 3 genes down-regulated in ZSA) (Supplementary Table S7), Photosynthesis-antenna proteins (Os00300), with 4 genes down-regulated in ZSA, alpha-Linolenic acid metabolism (Os00592), with 10 genes (8 genes up-regulated and 2 genes down-regulated in ZSA) (Supplementary Table S8) were the major enriched pathways among the DEGs.

### Confirmation of gene expression patterns by qRT-PCR

**Figure 7:**
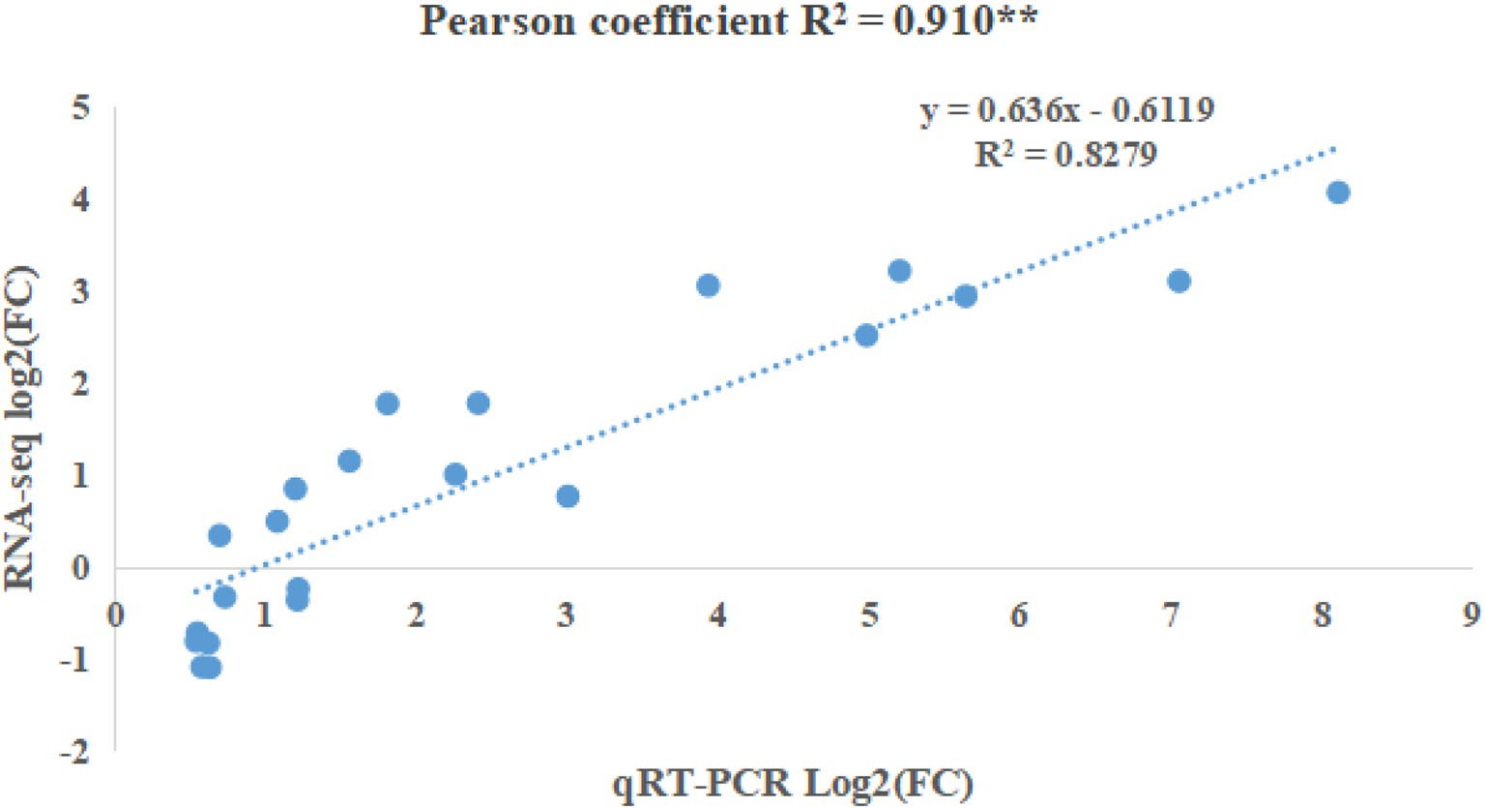
Linear regression between the levels of qRT-PCR data and transcript expression.

The expression patterns of randomized eight candidate genes that related to phenylpropanoid biosynthesis, starch and sucrose metabolism, alpha-Linolenic acid metabolism, and JA biosynthesis were examined by qRT-PCR to validate the RNA-sequencing results (Fig. 7; Supplementary Table S9). The expression patterns determined by qRT-PCR were compared with those of the RNA-seq assay. The qRT-PCR and RNA-seq results showed a positive correlation coefficient (Pearson coefficient R^2^ = 0.910), indicating that the transcriptome data were able to reflect transcript abundance in our study.

### DAM analysis of ABA pretreatment in response to salinity stress

**Figure 8:**
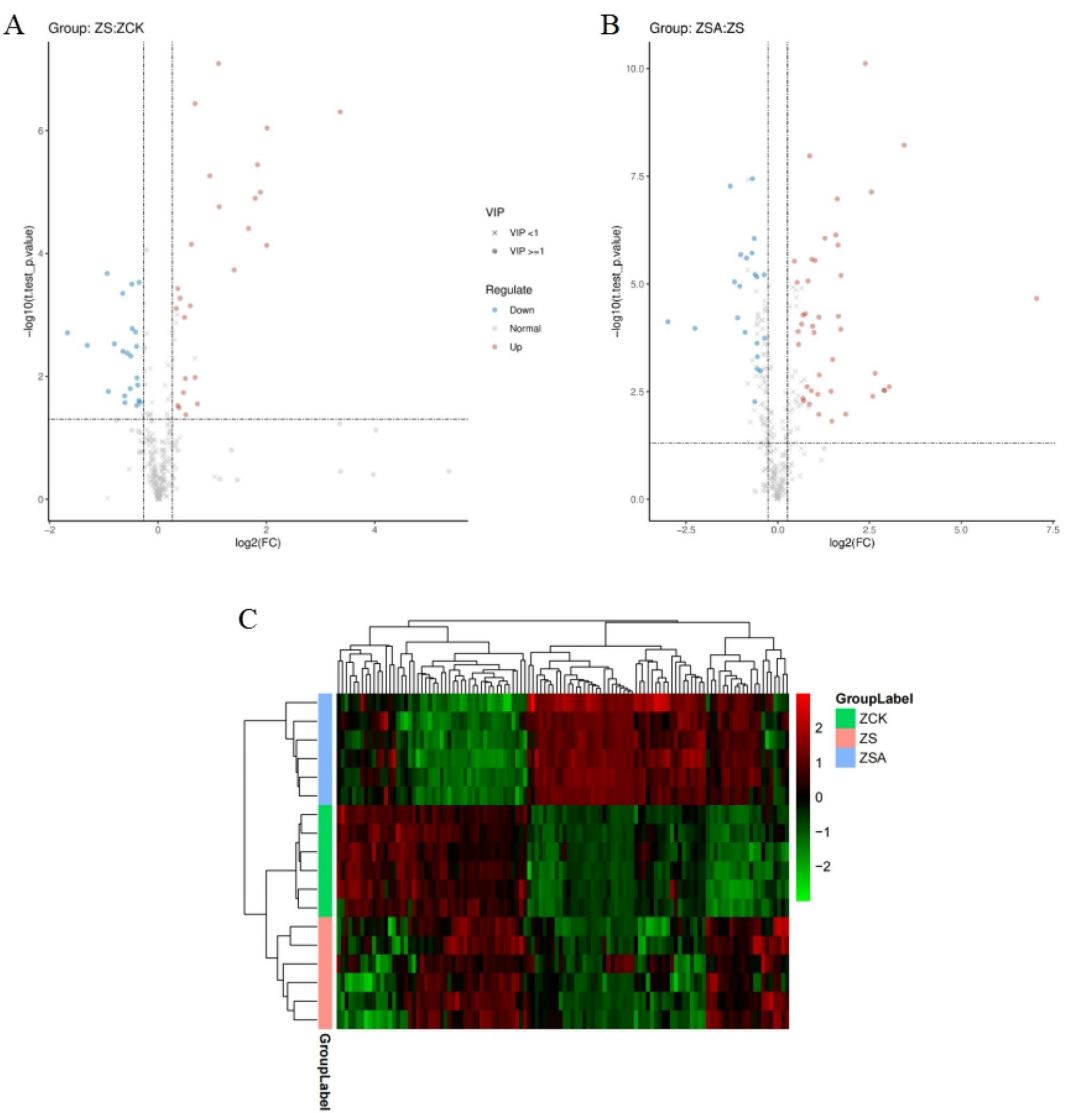
Volcano plot of different metabolites screened for different comparison groups (**A**,**B**). The ordinate log_t.test_p.value represents the P-value value of the-log10 for the *t* test, and the abscissa log_2_FC represents the log2 for the value of the FC of the difference multiple. Heatmap of the differential metabolites for the different comparison groups (**C**). The color from green to red indicates the expression abundance of metabolites from low to high phy.per.

We performed metabolite profiling of rice samples (ZCK, ZS, and ZSA) to assess the overall metabolic effects of pretreatment with ABA on rice seedling leaves under salt stress. Defined the metabolites that differential fold change (FC) ≥ 1.2 or FC ≤ 0.83 and Variable Importance in the Projectior (VIP) ≥ 1 as differential metabolites. 47 differential metabolites were detected in ZCK vs. ZS, with 25 up-regulated and 22 down-regulated, the levels of 1-(3,5-Dihydroxyphenyl)-12-hydroxy-2-tridecanyl acetate, Tetranor-12r-hete and 4-Benzyl-N-(3,5-dimethyl-4-isoxazolyl) tetrahydro-1(2H)-pyrazinecarboxamide, among others, increased at least two fold, whereas D-Sphingosine, 2-Naphthalenesulfonic acid, Rehmannioside C and 3,4,5-Trimethoxycinnamic acid, among others, decreased under salt stress. Between the ZS and ZSA treatments, 63 differential metabolites were detected, with 42 up-regulated and 21 down-regulated; Cyclamic acid, 12-Hydroxydodecanoic acid, 1-(1-Hydroxybutyl)-1,3,4,5,6,7-hexahydro-2-benzofuran-4,5,6,7-tetrol and Azelaic acid, among others, showed relatively high levels in ABA + NaCl treatment, whereas Eriodictyol, Glycerophospho-N-palmitoyl ethanolamine, Leucoside, among others, showed relatively low levels (Fig. 8) (Supplementary Table S10).

All the differential metabolites were subjected to hierarchical clustering (Fig. 8C), we could obviously detected the differential accumulated between the two comparison groups.

**Figure 9:**
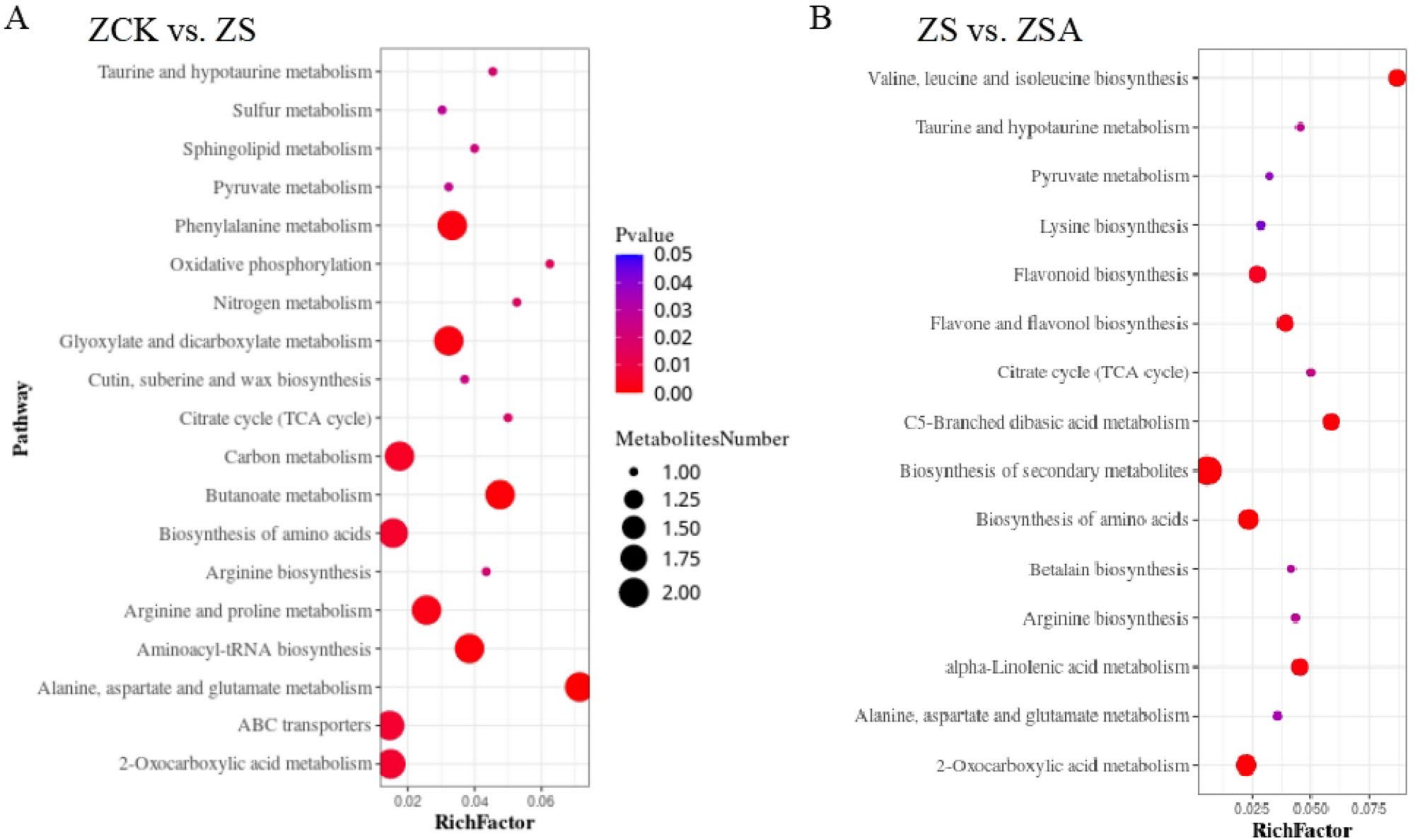
Functional analysis of DAMs based on KEGG pathway annotations. Pathways with a Q-value ≤ 0.05 that were significantly enriched in DAMs between the control and NaCl group (**A**), NaCl and NaCl + ABA treatment group (**B**) after 72 h of foliar sprayed ABA, were analyzed with the KEGG database.

Complex metabolic responses in organisms are generally influenced and regulated by multiple genes and proteins, ultimately leading to systemic changes in the metabolome. To identify the main pathways involved in the leaves of rice seedlings under salt stress that response to ABA, we mapped the DAMs to KEGG pathways and the metabolic pathway enrichment results were obtained (Fig. 9). 47 significant DAMs in ZCK vs. ZS were assigned to 28 KEGG pathways (Supplementary Table S11), including Alanine, aspartate and glutamate metabolism (00250), Butanoate metabolism (00650), Metabolic pathways (01100), Aminoacyl-tRNA biosynthesis (00970), Phenylalanine metabolism (00360), Glyoxylate and dicarboxylate metabolism (00630), Arginine and proline metabolism (00330), Carbon metabolism (01200), Biosynthesis of amino acids (01230) and 2-Oxocarboxylic acid metabolism (01210), among others. In ZS vs. ZSA, 63 significant DAMs were assigned to 16 KEGG pathways (Supplementary Table S12), including Metabolic pathways (01100), Valine, leucine and isoleucine biosynthesis (00290), Biosynthesis of amino acids (01230), Biosynthesis of secondary metabolites (01110), 2-Oxocarboxylic acid metabolism (01210), C5-Branched dibasic acid metabolism (00660), alpha-Linolenic acid metabolism (00592), Flavone and flavonol biosynthesis (00944) and Flavonoid biosynthesis (00941), among others.

### ABA regulated oxidation-related gene expression to protect rice leaves from salt stress

Antioxidant enzymes could protect plants against oxidative damage induced by stress. In our study, some redox-related genes were detected, including glutathione hydrolase, glutathione S-transferase, peroxidase and hydrogen peroxidase, which participate in the removal of ROS. In ZCK vs. ZS, 11 genes were up-regulated in ZS, in contrast, 30 genes (*Os01g0372400*, *Os01g0374000*, *Os09g0367700*, *Os10g0527400*, *Os04g0651000*, *Os11g0112200*, particularly) were up-regulated and 3 genes (*Os03g0368900*, *Os01g0151400*, *Os01g0151500*) were down-regulated in ZSA. 18 genes (10 genes down-regulated and 7 genes up-regulated in ZS, 17 genes up-regulated and 1 gene down-regulated in ZSA) were also assigned to hydrogen peroxide catabolic process (GO:0042744) (Supplementary Tables S5, 6 and S13).

### Differential expression of starch and sucrose metabolism genes

Sucrose and starch are widely distributed in various plant tissues, and they are important transportation and storage substances of plant carbohydrate, especially their mutual transformation. All starch and sucrose genes expression levels were almost significantly down-regulated after salinity stress without ABA treatment, including five genes associated with glucan endo-1,3-beta-glucosidase 3, one trehalose 6-phosphate phosphatase and two probable alpha, alpha-trehalose-phosphate synthase [UDP-forming] 10. Conversely, ABA treatment prominently up-regulated these genes in the presence of salinity. Besides, we detected two sucrose synthase genes, sucrose synthase 7 and 5, which were obviously up-regulated in ZS vs. ZSA (Table 4), indicated that exogenous ABA enhanced the synthesis and accumulation of sucrose in photosynthesis process, which contributes to maintain normal growth under salt stress.

**Table 4:** Expression levels of genes associated with starch and sucrose metabolism in rice seedling.

Gene_ID	Annotation	Expression level (log_2_FC)		
		ZCK vs. ZS	ZCK vs. ZSA	ZS vs. ZSA
Os01g0713200	Glucan endo-1,3-beta-glucosidase gii	− 2.29	0.69	2.98
Os06g0676700	Probable alpha-glucosidase os06g0675700	− 3.77	− 1.16	2.61
Os07g0539900	Glucan endo-1,3-beta-glucosidase 3	− 1.72	1.39	3.12
Os12g0505800	Trehalose 6-phosphate phosphatase (TPP)	− 6.08	0.52	6.59
Os01g0720600	Probable starch synthase 4, chloroplastic/amyloplastic	− 0.49	1.11	1.60
Os01g0940700	Glucan endo-1,3-beta-glucosidase, acidic isoform	− 0.69	2.24	2.93
Os01g0940800	Glucan endo-1,3-beta-glucosidase GII	− 0.02	1.09	1.12
Os04g0249500	Sucrose synthase 7	− 0.06	1.62	1.68
Os04g0309600	Sucrose synthase 5	− 0.23	0.87	1.11
Os04g0474800	Beta-glucosidase 12	− 0.28	0.76	1.05
Os05g0128900	Probable alpha,alpha-trehalose-phosphate synthase [udp-forming] 10(tps)	− 1.92	1.67	3.60
Os05g0153500	Probable alpha,alpha-trehalose-phosphate synthase [udp-forming] 10	− 3.21	2.19	5.42
Os05g0580000	Glucose-1-phosphate adenylyltransferase large subunit 3, chloroplastic/amyloplastic-like	0.76	2.87	2.11
Os06g0131500	Glucan endo-1,3-beta-glucosidase 14	NA	5.13	5.18
Os07g0412100	Granule-bound starch synthase 1b, chloroplastic/amyloplastic	0.11	− 1.12	− 1.23
Os07g0539100	Glucan endo-1,3-beta-glucosidase 3	− 1.37	2.11	3.48
Os07g0539300	Glucan endo-1,3-beta-glucosidase 12	− 0.21	1.08	1.29
Os07g0539900	Glucan endo-1,3-beta-glucosidase 3	− 1.72	1.39	3.12
Os07g0543100	Beta-amylase	− 0.11	− 1.24	− 1.13
Os08g0244500	Glucan endo-1,3-beta-glucosidase 7	− 0.59	1.22	1.81
Os08g0414700	Probable alpha,alpha-trehalose-phosphate synthase [udp-forming] 10	− 0.92	1.02	1.94
Os09g0491100	Beta-glucosidase 30	− 0.23	2.83	3.06
Os09g0511900	Probable inactive beta-glucosidase 33	0.34	− 1.04	− 1.37
Os10g0370500	probable glucan 1,3-beta-glucosidase A	− 0.34	0.80	1.15

### ABA regulated genes and metabolites involved in Flavonoid biosynthesis and JA biosynthesis to promote rice leaf development

**Figure 10:**
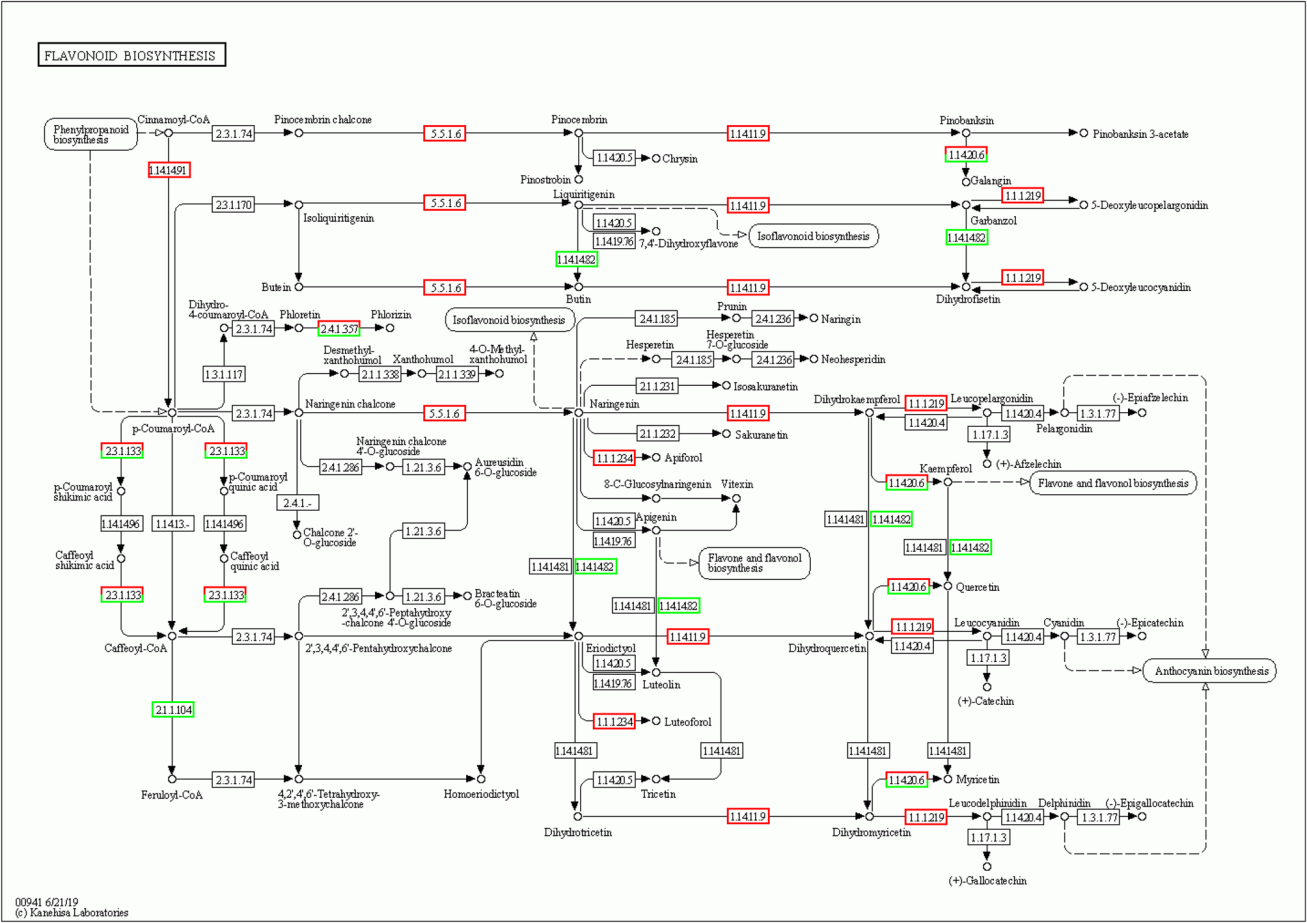
Coordinate up and down-regulation of genes involved in Flavonoid biosynthesis. The transcripts in Flavonoid biosynthesis pathway were up-regulated in ZS vs. ZSA. Green rectangle boxes indicated down-regulated genes and the red indicated up-regulated.

A combined transcriptomic and metabolic analysis can better explain the transcriptional regulation of metabolic pathways. We simultaneously mapped differentially expressed genes and metabolites from the same treatment groups to KEGG pathways to elucidate the relationships between genes and metabolites. Between ZS and ZSA, differentially expressed genes and metabolites were assigned to Flavonoid biosynthesis (00941) and alpha-Linolenic acid metabolism (00592). In the Flavonoid biosynthesis (00941) pathway, we detected 16 genes (with 13 genes up-regulated and 3 genes down-regulated) and two metabolites (Eriodictyol and Luteolin) in ZSA, specially, the gene *flavonoid 3'-monooxygenase CYP75B3-like*(*Os10g0320100*) and the levels of Eriodictyol and Luteolinwere down-regulated (Supplementary Tables S7; S10 and Fig. S1; Fig. 10). Additionally, the genes *probable 2-oxoglutarate-dependent dioxygenase At5g05600*, *flavanone 3-dioxygenase 2-like* (*Os03g0289800*, *Os04g0581000* and *Os05g0127500*) that catalyse an early step in the flavonoid biosynthesis pathway leading to the production of flavanols and anthocyanins, which were all up-regulated in ZSA, indicating that ABA may mitigate the effect caused by salinity via stimulating the flavonoid biosynthesis pathway.

**Figure 11:**
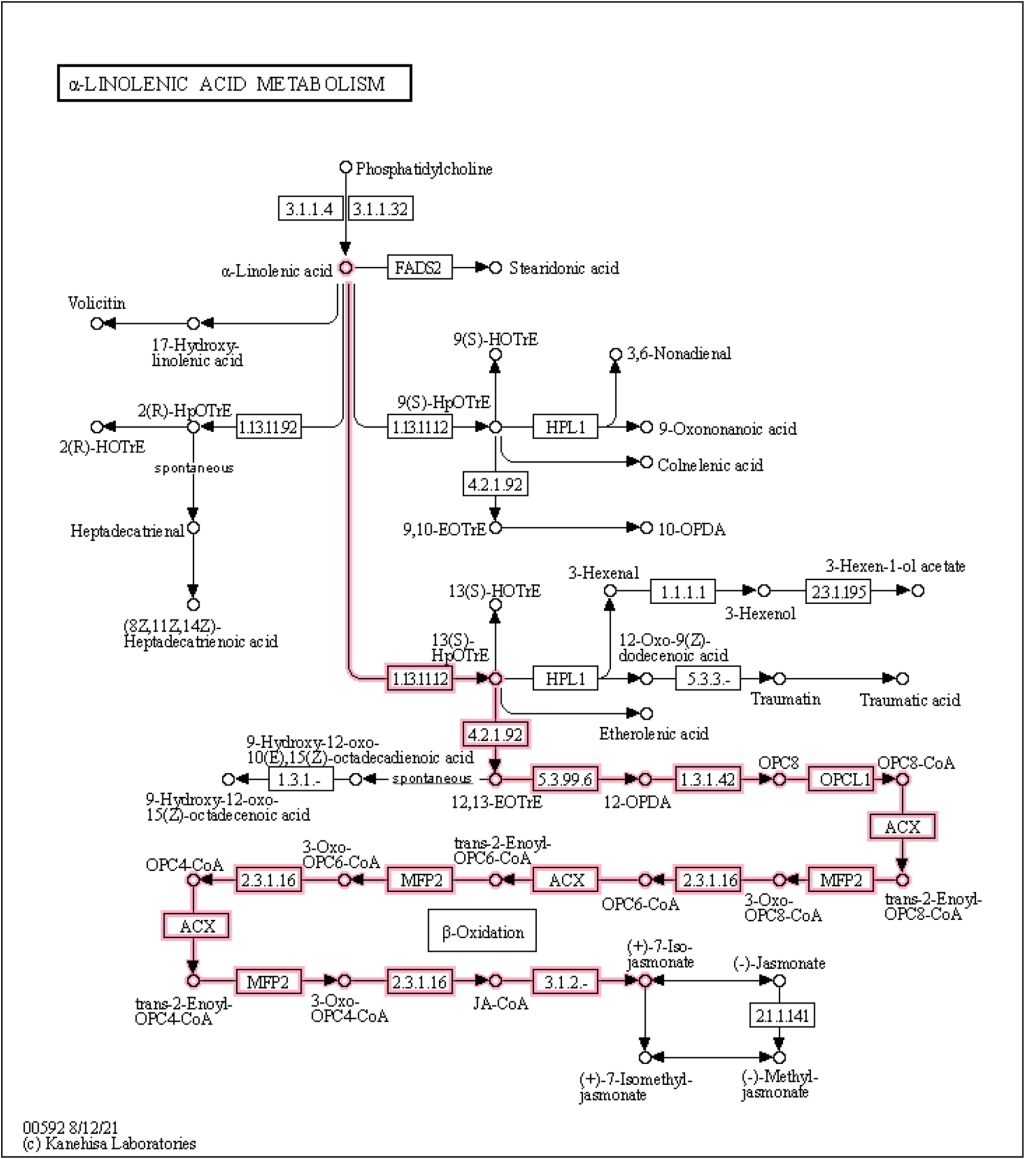
Coordinate the metabolites involved in JA biosynthesis pathway. The bright line and frame represent the synthetic pathway of JA in the metabolism patghway of α-Linolenic acid.

In the alpha-Linolenic acid metabolism (00592) pathway, we detected 8 genes up-regulated, 2 genes (*Os02g0194700*, *Os10g0562200*) down-regulated in ZSA, as well as two metabolites (12-Oxo phytodienoic acid, 13(S)-HOTrE) levels were up-regulated (Fig. S2; Supplementary Tables S8 and S10), suggested that *lipoxygenase 2.3, chloroplastic* (EC:1.13.11.12) negatively regulated the 13(S)-HOTrE level, and the gene *chloroplast envelope quinone oxidoreductase homolog* (*Os04g0372700*) positively regulated the 12-Oxo phytodienoic acid level. Jasmonic acid (JA) are a class of fatty acid derivatives that are ubiquitous in plants. As signaling molecules, JA can not only effectively mediate plant defense responses to pathogens, herbivores and abiotic stresses, but also regulate plant growth and development, such as root elongation, carbohydrate accumulation and fruit ripening. In JA biosynthesis pathway (Fig. 11), we detected that *phospholipase A1-Ibeta2, chloroplastic* gene (*Os10g0562200*), which catalyzed the reaction of phosphatidylcholine into alpha-Linolenic acid, were down-regulated, as well as JA biosynthesis-related gene *lipoxygenase 2.3, chloroplastic* (*Os02g0194700*). However, we detected another six JA biosynthesis-related genes (putative 12-oxophytodienoate reductase 1–6, *Os06g0215500*, *Os06g0215600*, *Os06g0215900*, *Os06g0216000*, *Os06g0216200*, *Os06g0216300*) were up-regulated (Supplementary Table S8), indicating that ABA may mitigate the effect caused by salinity stress via stimulating the lipid metabolism and regulate JA biosynthesis pathway.

## Discussion

High salinity causes impaired plant metabolism and physiological activities due to reduced osmotic potential, ion toxicity and nutritional imbalance, leading to organelle damage, disruption of photosynthesis, respiration, and protein synthesis^8^. Therefore, increased plant stress resistance and defense mechanisms is critical for sustainable crop production in salt-affected areas. An important role of ABA in a wide variety of plants is to improve the tolerance to stresses such as drought, salinity, cold, and heat^9–11^, which is considered an important signaling molecule that can trigger signaling and regulatory mechanisms to adverse stress. The molecular mechanisms underlying plant responses to ABA treatment under salinity are poorly understood. To characterize the enriched pathways and genes affected during salt and ABA treatment, we performed transcriptomic and metabolomic analyses in rice leaves. We identified DEGs and DAMs in salt-treated and ABA-treated rice leaves and investigated significantly enriched biological processes and pathways by GO and KEGG analysis. The present findings suggested that many phenylpropanoid biosynthesis-related genes, Flavonoid biosynthesis-related genes, and alpha-Linolenic acid metabolism-related genes involved in ABA signaling pathway in salt-stressed rice leaves. Similar results were obtained by Yang et al.^3^. Recently, more and more studies have pointed out that plant secondary metabolites involved in plant defense response to abiotic stress. Previous study has demonstrated that some angiosperms can accumulate flavonoids and flavonol glycosides, responding to UV stress by absorbing UV-B, as well as accumulate anthocyanins to combat drought, cold, high light, and other stresses^12,13^.

### Role of antioxidant systems in salinity stress

One of the molecular effects of compounds that enhance plant tolerance to abiotic stresses involves the activation of antioxidant processes. In the present study, we found some genes encoding key enzymes such as CAT, POD, S-transferase gene (GST), ascorbate peroxidase gene and glutathiredoxin (GRX), which were contributed to remove excess O_2_^–^ and hydrogen peroxide and ensure plant survival^14^. Xie et al.^15^ found that 10 GSTs and 2 peroxidase genes were up-regulated in citrus roots, which participated in the salt resistance study of citrus roots. In the present study, we detected 7 and 10 peroxidase genes (including peroxidase 1, cationic peroxidase SPC4, peroxidase 70, etc.) were up-regulated and down-regulated, respectively under salt stress (Supplementary Table S6). However, most peroxidase genes were down-regulated in rice leaves under salt stress, which was opposite to some findings, this may be due to the fact that most of the peroxidase genes found in these studies belong to different members of the same gene family^16^. On the other hand, peroxidase belongs to a large gene family with more than 70 members in a plant species. Their expression levels and patterns were different in different tissues^17^. The down-regulated peroxidase genes may be due to that *Huanghuazhan* could reduce oxidative stress, or possibly because the up-regulated expression of these genes appears at post-transcriptional levels. Similar results were also obtained in the salt-sensitive rice variety IR29^18^ and IR64^19^. All of these results suggested that the antioxidant enzymes play an important role in multiple developmental processes. Additionally, we detected a blue copper protein (*Os08g0482700*), which is a ROS-scavenging signaling molecule and contributed to preventing the formation of a highly toxic OH-^14^, was up-regulated in ABA + NaCl treatment, all of these suggested that a strict regulation of the oxidative stress pathway to the salt stress response, and ABA could support the rice adaptation to salt stress via maintaining the redox status and improving salinity tolerance.

### Role of flavonoids in salinity stress

Flavonoids are synthesized through the phenylpropanoid pathway to convert phenylalanine to 4-coumaroyl-CoA and eventually enter the flavonoid biosynthesis pathway. Our transcriptome results showed that the genes relate to phenylpropanoid pathway (00940): 4-coumarate-CoA ligase 2 (*Os02g0697400*) and 4-coumarate-CoA ligase 5 (*Os08g0448000*) were up-regulated under salt stress, which contributed to enhancing flavonoid biosynthesis (00941). Transcriptome analysis of the roots and stems of rice showed that salt stress significantly affected the metabolism of carbohydrates and amino acids, and induced the accumulation of secondary metabolites-related genes, phenolic salts, and flavonoids in roots^20^. Chandran et al.^20^ found that secondary metabolites-related genes such as flavonoid accumulation in rice roots could be induced under salt stress. In the present study, we detected 13 flavonoid biosynthesis-related genes (e.g. *cytochrome P450 CYP73A100*, *probable 2-oxoglutarate-dependent dioxygenase*, *flavanone 3-dioxygenase 2-like*, *protein DMR6-LIKE OXYGENASE 1*, etc.) were up-regulated in ABA + NaCl treatment, which is consistent with the result of qRT-PCR. Additionally, the up-regulation of *cytochrome P450 CYP73A100* expression indicated that these proteins are required for the biosynthesis of several important compounds, such as hormones, defense-related products, and fatty acids^21^. Similar result was obtained in previous study^22^. Han et al.^23^ indicated that these up-regulated genes (*cytochromeP450 76AD1*, *cytochromeP450 84A1*, *cytochromeP450 CYP73A100*) may be involved in ABA signaling, implied that ABA signaling may be functionally important and exhibits a rapid response to Suaeda rigida early under salt stress. Exogenous ABA application prominently up-regulated *cytochrome P450 CYP73A100*, which may contribute to energy recovery, repair of cell membranes, and fatty acid synthesis.

It should be noted that we detected two important metabolites (Luteolin and Eriodictyol) were differentially accumulated. El-Shafey and AbdElgawad^24^ demonstrated that Luteolin could enhance the resistance of salt stress by enhancing the antioxidant systems in maize. In addition, the up-regulation of Flavonoid 3’hydroxylase (F3´H) expression in flowers of Taraxacum antungenseis consistent with the trend of Luteolin accumulation^25^. In the present study, the metabolic analysis showed that some DAMs were assigned to flavonoid biosynthesis (00941), and we detected that the gene *flavonoid 3’-monooxygenase CYP75B3-like* (*Os10g0320100*) and the levels of Eriodictyol and Luteolin were all down-regulated in ABA + NaCl treatment. Similar result was obtained by Song et al.^26^, who demonstrated that exogenous melatonin promoted Luteolin biosynthesis in pigeon pea through the CcPCL1 and CcF3´H-5 pathways under salt stress, and over-expression CcPCL1 and CcF3´H-5 in transgenic plants greatly improved resistance of salt stress by promoting Luteolin biosynthesis. Hence, we speculate that ABA enhanced rice tolerance to salt stress might mediate the accumulation of Luteolin and Eriodictyol through the gene *flavonoid 3’-monooxygenase*, *CYP75B3-like* (Os10g0320100), and this will be a focus of our future research.

### Role of JA and α-linoleic acid pathway in salinity stress

Jasmonic acids (i.e. cis-OPDA, JA, JA-Ile, and methyl jasmonate) are the major phytohormone involved in the stress response^27^. In the present study, we detected six JA biosynthesis-related genes, putative *OPR1* (Os06g0215500), *OPR2* (Os06g0215600), *OPR3* (*Os06g0215900*), *OPR4* (*Os06g0216000*), *OPR5* (*Os06g0216200*), *OPR6* (*Os06g0216300*), which were all up-regulated in ABA treatment under salt stress, consistent with the confirmation of gene expression patterns by qRT-PCR, indicating that ABA may mediate the biosynthesis and signal transduction of JA plant hormones to promote the growth of rice under salt stress. Wang et al.^6^ showed that exogenous ABA significantly up-regulated genes related to lipid and fatty acid metabolism and cytoplasmic transport in the aerial parts of two genotypes of rice under salt stress, indicating that enhanced lipid metabolism may also contribute to improving salt tolerance in rice^6^. From the metabolic analysis, we detected the levels of two differentially metabolisms (12-OPDA and 13(S)-HOTrE) in the alpha-Linolenic acid metabolism (00592) pathway, which were regulated by *lipoxygenase 2.3, chloroplastic* (EC:1.13.11.12) and *chloroplast envelope quinone oxidoreductase homolog* (*Os04g0372700*), respectively, were significantly up-regulated in ABA + NaCl treatment, indicating that ABA may mitigate the effect caused by salinity stress via stimulating the lipid metabolism. On the other hand, some researches showed that JA synthesis-related genes were down-regulated in sweet Sorghum under salt stress^28^, or the negative expression of regulators of JA and ETH signaling was significantly up-regulated in S.alopecuroides root tissues, indicating that JA may have been a negative regulatory in S.alopecuroides roots in response to salt stress^29^. All of these results were also similar to our transcriptome analysis results. Additionally, the wheat *OPR 1* gene (*TaOPR1*) has been shown to increase plant tolerance to high-salt stress by increasing MYC2 expression, thereby activating ABA-dependent signaling pathways^30^. All of these suggested that JA-biosynthesis related genes may play a key role in increasing salt tolerance, and this will be a focus of our future research.

### Role of trehalose in salinity stress

Previous studies have shown that the trehalose pathway plays an important role in regulating the usage and distribution of sucrose, coordinating the source-reservoir relationships, and improving the effective utilization of carbohydrates^31^ and increased crop yield^32^. Overproduced trehalose has considerable potential to improve abiotic stress tolerance in transgenic rice plants, which showed higher levels of tolerance to salt, drought, and cold stress as compared with untransformed controls^33^. In higher plants, trehalose is catalyzed by two enzymes: trehalose-6-phosphate synthase (TPS) and trehalose-6-phosphate phosphatase (TPP). In a previous report, the expression levels of *OsTPP1* and *OsTPP2* were induced by exogenous ABA, which can improve the stress tolerance in rice^34,35^. Rahman et al.^36^ showed that *OsTPP2*, *OsTPP3*, *OsTPP8* and *OsTPP9* were up-regulated under salt, drought and cold treatments, while *OsTPP1*, *OsTPP4*, *OsTPP5*, *OsTPP6*, and *OsTPP7* were down-regulated under salt stress. This clearly demonstrates their important role in abiotic stress tolerance. Additionally, *GhTPS11* in cotton^37^, over-expressing *IbMIPS1*^38^ in the sweet potato plants showed that ABA biosynthesis and signaling genes as well as trehalose synthesis-related genes were generally up-regulated. In our study, we detected that the expression levels of 3 *TPS10* (*Os05g0128900*, *Os05g0153500*, *Os08g0414700*) and one *TPP* gene (*Os12g0505800*) associated with starch and sucrose metabolism pathway were down-regulated under salt stress, while ABA treatment significantly up-regulated these genes under salt stress. This may be due to that ABA is involved in protecting the plant from abiotic stress through osmoregulation, detoxification of ROS and stabilization of the quaternary structure of proteins^39^. Previous study showed that the cucumber genes were expressed in roots, stems and leaves, however, they were varied in both levels, indicating that the *CsTPS* genes were tissue-specific. In addition, *CsTPS1* and *CsTPS6* were found to be down-regulated upon salinity treatment, and reached the lowest expression levels at 24 h^40^. Thus, we speculate that the differentially expression levels of trehalose-related genes are related to different tissues. Vishal et al.^41^ detected that *OsTPS8* may regulate suberin deposition and trehalose via ABA signaling, thus conferring salt tolerance in rice. These studies suggested a close relationship between trehalose and ABA in stress tolerance and growth and development.

## Conclusion

Salinity stress adversely affects the growth and development of rice, and ABA could increase its tolerance to salt stress. Compared with ZCK vs. ZS, DEGs specific to ZS vs. ZSA was significantly enriched in carbohydrate metabolism. Exogenous ABA obviously increased the expression of starch and sucrose genes (sucrose synthase 5 and sucrose synthase 7), which contributed to the synthesis and accumulation of sucrose in photosynthesis process under salt stress. Some redox-related genes including glutathione hydrolase, glutathione S-transferase, peroxidase and hydrogen peroxidase, as well as many differential metabolites (particularly Cyclamic acid) that belong to organic acid and derivatives or lipid and lipid-like molecules, were also up-regulated in ZS vs. ZSA when compared with ZCK vs. ZS, which mitigated oxidative damage. In addition, ABA could enhance rice leaves growth and development by regulating flavonoid biosynthesis and linoleic acid metabolism pathway. ABA decreased the level of Eriodictyol and Luteolin and up-regulated the expression of six JA biosynthesis-related genes. ABA also altered the expression of other genes related to glutathione metabolism, phenylpropanoid biosynthesis, flavonoid biosynthesis, photosynthesis-antenna proteins and alpha-Linolenic acid metabolism. In conclusion, the results in this study elucidated that exogenous ABA alleviated salinity toxicity and enhanced rice leaf development via mediating expression of genes in different pathways, however, candidate genes need to be further verified for their function.

## Materials and methods

### Plant growth and treatment

In the current study, seeds of inbred rice variety *Huanghuazhan* (Z) were used as the plant materials. The seeds were supplied by Guangdong Tianhong Seed Company Limited, Zhanjiang and the ABA solution was provided by Sichuan Lomon Fusheng Technology Co., Ltd. Healthy and uniform seeds were sorted manually and sterilize with 2.5% sodium hypochlorite solution for 15 min, then thoroughly rinse with distilled water. 75 seeds were germinated in the dark for 2 days at 30 ℃ using plastic pots (19.5 × 14.5 × 17.5 cm) containing 3 kg of substrates (Latosol: sand = 3:1, v/v). The relative humidity was maintained at 75% throughout the experiment. Thirty pots per experimental plot were arranged in a split plot scheme in a completely randomized block design with three replications, resulting in a total of 90 pots. After 8 d of germination, when rice seedlings grew to two leaves and one heart, 5 mg L^−1^ ABA were applied to leaves as foliar spray until the leaf surface was moist^4^. Salt treatment was initiated to the plants at the same time by adding NaCl at a rate of 25 mM increment per day, to reach a final concentration of 50 mM in the solution. The other treatments included: (i) control (ZCK): 0 mg L^−1^ ABA + 0 mM NaCl, (ii) (ZS): 0 mg L^−1^ ABA + 50 mM NaCl, (iii) (ZSA): 5 mg L^−1^ ABA + 50 mM NaCl. The leaves samples were collected after 24 h of all salt stress treatments with three biological replicates, including 4 pots of sample per biological replicate. And then immediately frozen in liquid nitrogen and stored at − 80 °C for further laboratory analysis.

### RNA extraction and transcriptome sequencing

According to protocol the total RNA was extracted using Trizol reagent kit (Invitrogen, USA). RNA quality and concentration were assessed by Agilent 2100 Bioanalyzer (Agilent Technologies, Palo Alto, CA, USA). Eukaryotic mRNA was enriched by Oligo (dT) beads, after RNAs fragmentation. The enriched mRNA was fragmented into short segments using fragmentation and reverse transcribed into cDNA with random primers. Second-strand cDNA were synthesized by DNA polymerase I, RNase H, dNTP and buffer. Rolling cycle amplification was used to duplicate DNA molecules in the PCR reaction. DNA nanoball (DNB) was generated which contain multiple copies of DNA. Sufficient quality DNBs loaded into patterned nanoarrays using high-intensity DNA nanochip technique and sequenced through combinatorial Probe-Anchor Synthesis (cPAS). Sequenced using Illumina HiSeq2500 by BGI-Shenzhen (Shenzhen, China).

### Sequence assembly, annotation and classification

The sequencing data were processed by SOAPnuke^42^ by (1) Removing reads containing sequencing adapter; (2) Removing reads whose low-quality base ratio (base quality less than or equal to 15) is more than 20%; (3) Removing reads whose unknown base (‘N’ base) ratio is more than 5%, afterwards clean reads were obtained and stored in FASTQ format. The subsequent analysis and data mining were performed on Dr. Tom Multi-omics Data mining system (https://biosys.bgi.com). The clean reads were mapped to the reference genome using HISAT2^43^. After that, Ericscript (v0.5.5)^44^ and rMATS (V3.2.5)^45^ were used to detect fusion genes and differential splicing genes (DSGs), respectively. Bowtie2^46^ was applied to align the clean reads to the gene set, a database built by BGI (Beijing Genomic Institute in ShenZhen), in which known and novel, coding and noncoding transcripts were included. The Expression level of gene was calculated by RSEM (v1.3.1)^47^. The heatmap was drawn by pheatmap (v1.0.8)^48^ according to the gene expression difference in different samples. Essentially, differential expression analysis was performed using the DESeq2 (v1.4.5)^49^ or DEGseq^50^ or PoissonDis^51^ with Q value ≤ 0.05 (or FDR ≤ 0.001). To take insight to the change of phenotype, GO (http://www.geneontology.org/) and KEGG (https://www.kegg.jp/) enrichment analysis of annotated different expression gene was performed by Phy.per (https://en.wikipedia.org/wiki/Hypergeometric_distribution) based on Hypergeometric test. The significant levels of terms and pathways were corrected by Q value with a rigorous threshold (Q value ≤ 0.05)^52^.

### Validation of differential expression using qRT-PCR

To confirm the RNA-Seq results, 8 candidate DEGs were randomly validated by qRT-PCR. Based on the coding gene sequences, the primers of qRT-PCR were designed using primer premier 5.0 software, and β-actin genewas selected as the internal control^53^. The primers were designed and listed in Supplementary Table S1.The 2X SG Fast qPCR Master Mix (High Rox) (BBI, Canada, Inc) was used for the RT-qPCR assay and the RT-qPCR assays were carried out using the QuantStudio™ 1 Plus System (ABI/Thermo Fisher, USA). The relative targeted genes expression levels were calculated according to the 2^–ΔΔCt^ method^54^. Each sample was performed in three replicates.

### Extraction and quantification of metabolites

Metabolites were isolated from rice leaf tissues, with six replicates per treatment. After grinding and centrifugation, the supernatant was removed over the 0.22 μm filter membrane. The filtered samples were placed in the upper sample vial waiting for LC–MS analysis. A total of 20 μl from each sample was mixed into QC samples and used to assess the reproducibility and stability of the LC–MS analysis process. Sample analysis was performed on a Waters ACQUITY UPLC 2D (Waters, USA), coupled to a Q-Exactive mass spectrometer (Thermo Fisher Scientific, USA) with a heated electrosprayionization (HESI) source. Chromatographic separation was performed on a Hypersil GOLD aQ column (2.1 × 100 mm, 1.9 μm, Thermo Fisher Scientific, USA), with mobile phase A consisting 0.1% formic acid in water and mobile phase B consisting 0.1 formic acid in acetonitrile.The mass spectrometric settings for positive/negative ionization modes were as follows: spray voltage, 3.8/ − 3.2 kV; sheath gas flow rate, 40 arbitrary units (arb); aux gas flow rate, 10 arb; aux gas heater temperature, 350 °C; capillary temperature, 320 °C. The full scan range was 100–1500 m/z with a resolution of 70,000, and the automatic gain control (AGC) target for MS acquisitions was set to 1e6 with a maximum ion injection time of 100 ms. Top 3 precursors were selected for subsequent MSMS fragmentation with a maximum ion injection time of 50 ms and resolution of 30,000, the AGC was 2e5. The stepped normalized collision energy was set to 20, 40 and 60 eV.In order to provide more reliable experimental results, the samples were randomly sorted to reduce the system error. The QCs were injected at regular intervals (every 10 samples) throughout the analytical run to provide a set of data from which repeatability can be assessed.

### Metabolomic data analysis

Data were analyzed by fold change and T test. Fold Change (FC) was obtained by variation fold analysis and p-value were corrected by FDR (False Discovery Rate) to obtain q-value. We used a combination of the p-values of T-test with the VIP value of the OPLS-DA model to filter differentially accumulated metabolites, with additional screening criteria (p < 0.05, VIP > 1).

### Statistical analysis

The data were collated using WPS office software and SPSS 19.0 software for one-way ANOVA statistical analysis and the least significant difference (LSD) method for multiple comparisons, with significant differences defined at p < 0.05.

### Ethics approval and consent to participate

The study was performed in accordance with relevant institutional, national, and international guidelines and legislation.

### Supplementary Information

Supplementary Figures.

Supplementary Tables.

Supplementary Table S9.

Supplementary Table S10.

Supplementary Table S11.

Supplementary Table S12.

Supplementary Table S13.

## Data Availability

The datasets generated and/or analysed during the current study are available in the SRA repository, BioProject Accession (PRJNA931648). https://www.ncbi.nlm.nih.gov/sra/PRJNA931648.
