# Supplementary material for: Transcriptomic and metabolomic analyses reveal that ABA increases the salt tolerance of rice significantly correlated with jasmonic acid biosynthesis and flavonoid biosynthesis
Source: Sci Rep. 2023 Nov 21;13:20365. doi: 10.1038/s41598-023-47657-w (PMC10663488; doi:10.1038/s41598-023-47657-w)

Supplementary Fig. 1 Coordinate down-regulation of metabolites involved in Flavonoid biosynthesis. The metabolic profile in Flavonoid biosynthesis pathway were down-regulated in Z1 vs. Z2. Blue circle indicated down-regulated metabolites.

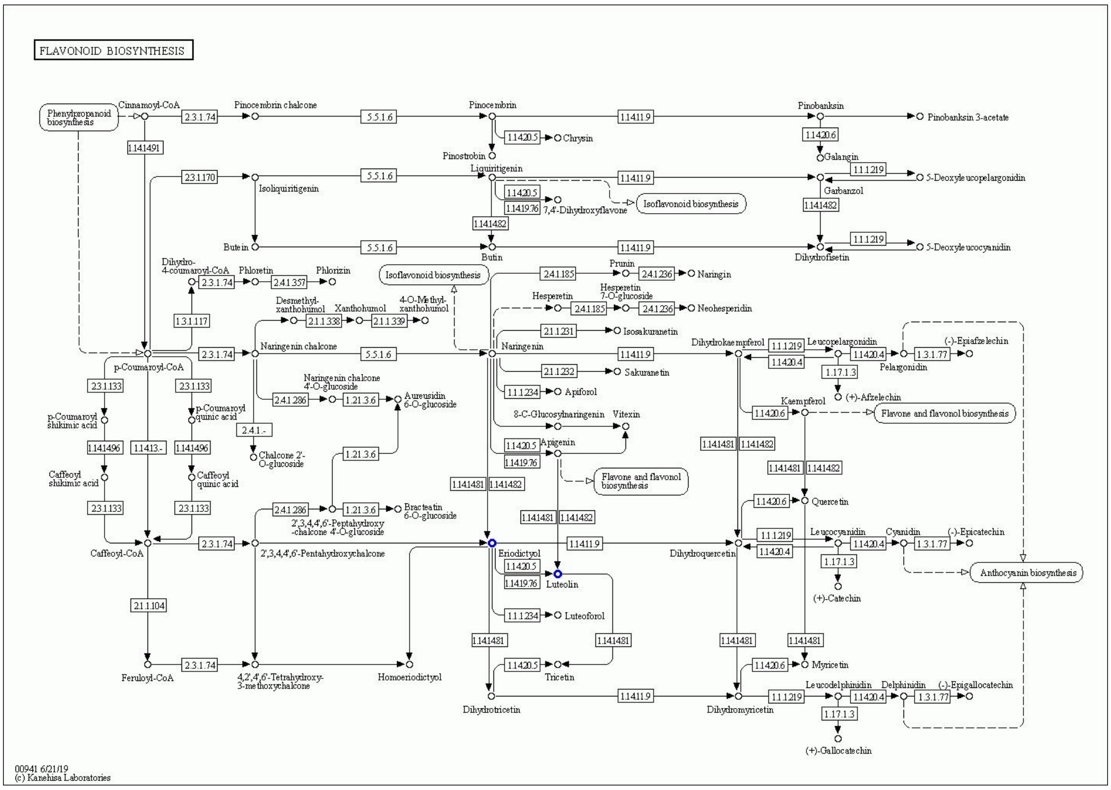

Supplementary Fig. 2 Coordinate up-regulation of metabolites involved in alpha-Linolenic acid metabolism. The metabolic profile in alpha-Linolenic acid metabolism pathway were up-regulated in Z1 vs. Z2. Red circle indicated up-regulated metabolites.

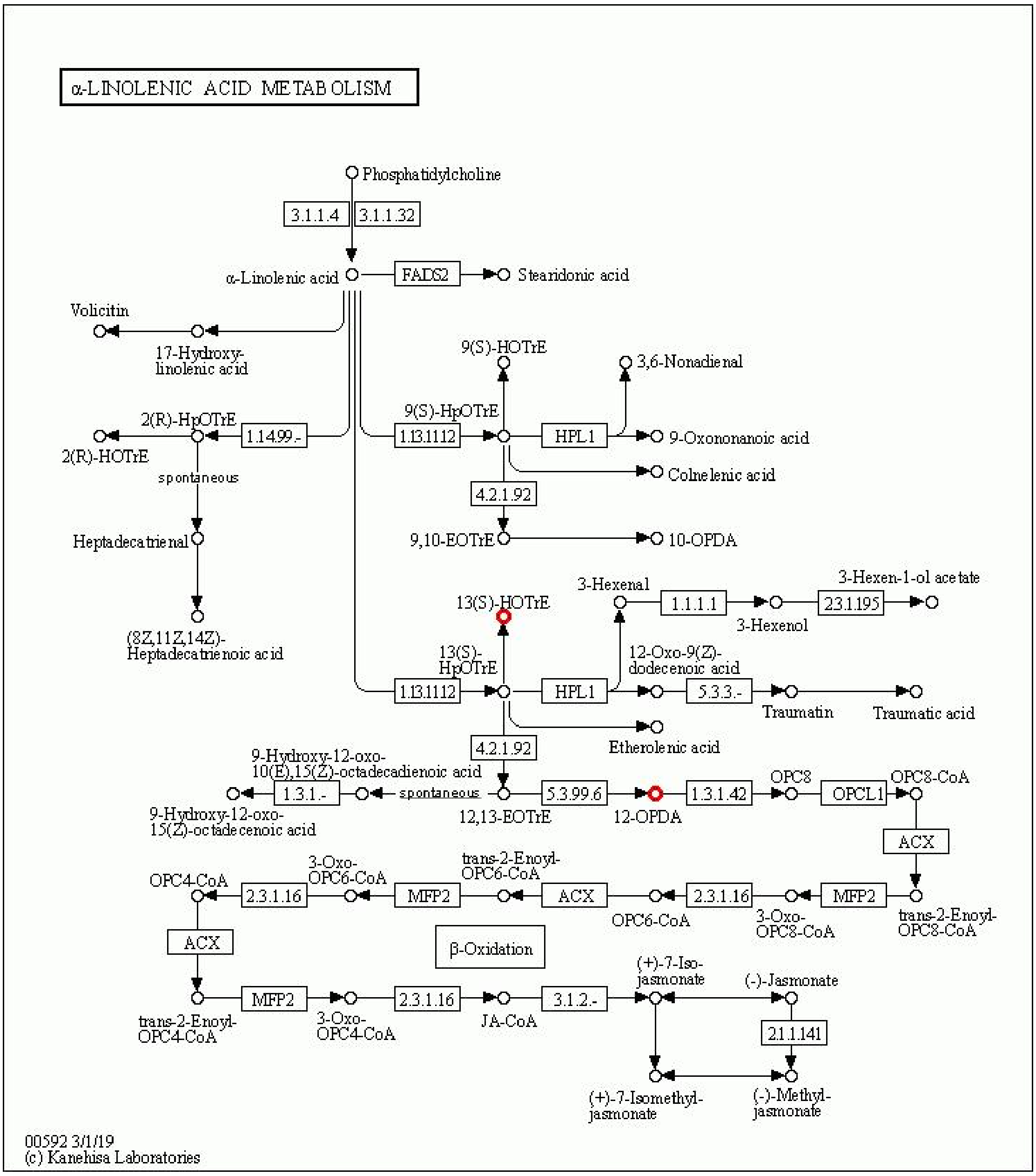

Supplement: Supplementary file 1 — Supplementary Figures. [file 41598_2023_47657_MOESM1_ESM.pdf]
