# Supplementary material for: Transcriptomic and metabolomic analyses reveal that ABA increases the salt tolerance of rice significantly correlated with jasmonic acid biosynthesis and flavonoid biosynthesis
Source: Sci Rep. 2023 Nov 21;13:20365. doi: 10.1038/s41598-023-47657-w (PMC10663488; doi:10.1038/s41598-023-47657-w)
Supplement: Supplementary file 2 — Supplementary Tables. [file 41598_2023_47657_MOESM2_ESM.pdf]

Supplementary Table S1 Primers used for qRT-PCR analyses.

Gene ID	Upstream primer	Upstream primer
Actin	AAAGGATGCCTATGTGGGTG	CTGTTGGCTTTGGGATTGAG
Os12g0111800	CCCACCATCCGAATAGCC	AGCACCGAGCCATCACAA
Os01g0327100	TTTGGCGAATGGGACGAT	GCGGCAGCAGCTTGTAGTAG
Os12g0505800	GAAGACGCTGGATTTCATAGTGT	AAATGCGGAGCTGGAGGT
Os05g0128900	AAGACGCTGGATTTCGTAGTG	GGAATGCAGAGCTGGAGGT
Os05g0153500	TCTCTCAGATGGAACAAAGCAG	GATGTAAACTTGCATGACCGG
Os04g0372700	GCAGCGACCATCAATCCA	GACCAACACCAGCAACCACT
Os06g0215600	GGGAGGAAGGCAACAAGG	TCTAAGCATCAGCAACCGAGT
Os06g0215900	AGGAAGGAAACAAGGTGATAGC	GCCCTCGTCTTTCTCATCAA

Supplementary Table S2 Summary of raw RNA-seq reads of rice seedling leaves under salt stress and ABA application

Sample	Raw Reads (M)	Clean Reads (M)	Clean Bases (Gb)	Clean Reads Q20 (%)	Clean Reads Q30 (%)	Clean Reads Ratio (%)	Mapped Ratio (%)	Unique Mapped Ratio (%)
Z0-1	50.68	44.80	6.72	95.36	88.27	88.40	83.18	81.53
Z0-2	48.93	44.67	6.70	95.21	87.93	91.29	83.88	81.21
Z0-3	48.93	44.80	6.72	95.10	87.59	91.56	83.63	81.95
Z1-1	47.55	43.81	6.57	95.28	88.03	92.12	84.28	82.60
Z1-2	48.93	44.92	6.74	95.20	87.87	91.79	84.16	82.46
Z1-3	48.86	44.81	6.72	95.30	88.09	91.71	84.01	82.35
Z2-1	44.48	40.93	6.14	95.08	87.59	92.02	83.91	82.20
Z2-2	44.02	40.53	6.08	94.92	87.14	92.07	84.32	82.60
Z2-3	48.93	44.83	6.72	95.23	87.84	91.62	84.37	82.63

Z0 represents non-stressed seedlings treated with water, Z1 represents 50 mM NaCl-stressed seedlings treated with water, Z2 represents 50 mM NaCl-stressed seedlings treated with 5 mg/L ABA.

Supplementary Table S3 Expression levels of genes associated with plant-pathogen interaction in rice seedling

Gene_ID	Annotation	Expression level (log_2FC)		
		Z0 vs. Z1	Z0 vs. Z2	Z1 vs. Z2
Os01g0382000	pathogenesis-related protein 1	-2.25	-0.49	1.76
Os01g0584900	WRKY transcription factor SUSIBA2	-1.72	-0.98	0.74
Os02g0609900	putative receptor-like protein kinase At3g47110	-2.41	-1.46	0.95
Os03g0133400	Chitin elicitor-binding protein	-1.03	-0.14	0.89
Os03g0335200	probable WRKY transcription factor 53	-2.82	-0.37	2.45
Os06g0287700	putative disease resistance RPP13-like protein 3	-1.74	-1.33	0.41
Os07g0129300	pathogenesis-related protein 1	-3.62	0.76	4.38
Os09g0417600	WRKY transcription factor WRKY76-like	-2.87	-0.89	1.98
Os09g0417800	WRKY transcription factor WRKY62	-2.29	-1.06	1.23
Os11g0228600	proline-rich receptor-like protein kinase PERK3	-1.09	-0.82	0.28
Os11g0229300	disease resistance protein RPM1	-2.15	-0.86	1.29
Os11g0229500	disease resistance protein RPM1	-1.19	-2.06	0.99

Supplementary Table S4 Expression levels of genes associated with Lysine biosynthesis and Monobactam biosynthesis in rice seedling

Gene_ID	Annotation	Expression level (log_2FC)		
		Z0 vs. Z1	Z0 vs. Z2	Z1 vs. Z2
Os03g0850400	aspartokinase 1, chloroplastic	-1.34	1.24	2.58
Os09g0294000	bifunctional aspartokinase/homoserine dehydrogenase 2, chloroplastic	-1.69	1.58	3.28

Supplementary Table S5 Expression levels of genes associated with glutathione metabolism in rice seedling

Gene_ID	Annotation	Expression level (log_2FC)		
		Z0 vs. Z1	Z0 vs. Z2	Z1 vs. Z2
Os01g0151400	glutathione hydrolase 3	0.07	-1.28	-1.34
Os01g0151500	glutathione hydrolase 3	-0.05	-1.20	-1.14
Os01g0372400	probable glutathione S-transferase GSTF1	-3.52	1.15	4.68
Os01g0374000	glutathione S-transferase 1	0.92	5.64	4.73
Os01g0692000	probable glutathione S-transferase GSTU6	0.34	2.03	1.69
Os01g0692100	probable glutathione S-transferase GSTU6	-0.07	1.14	1.22
Os02g0465900	gamma-glutamylcyclotransferase 2-2	-0.66	0.95	1.61
Os02g0600400	glucose-6-phosphate 1-dehydrogenase, cytoplasmic isoform	-0.23	0.89	1.12
Os03g0135100	glutathione S-transferase F11	-0.81	2.48	3.30
Os03g0283200	glutathione transferase activity	-0.29	0.94	1.23
Os06g0127900	ribonucleoside-diphosphate reductase small chain	-1.53	0.71	2.24
Os09g0367700	probable glutathione S-transferase GSTU1	2.16	7.16	4.99
Os09g0544400	glutathione transferase, catalytic activity	-0.71	0.96	1.67
Os10g0527400	probable glutathione S-transferase GSTU6	-0.05	5.52	5.57
Os10g0528300	glutathione transferase,Catalytic activity	0.05	2.67	2.63
Os10g0528400	glutathione transferase, Catalytic activity	-0.66	2.54	3.19
Os10g0529400	glutathione transferase, Catalytic activity	-0.23	1.24	1.47
Os10g0530900	Probable glutathione S-transferase GSTU6	-0.46	1.45	1.91

Supplementary Table S6 Expression levels of genes associated with phenylpropanoid biosynthesis in rice seedling

Gene_ID	Annotation	Expression level (log_2FC)		
		Z0 vs. Z1	Z0 vs. Z2	Z1 vs. Z2
Os01g0326300	peroxidase 1	-1.47	0.51	1.98
Os01g0327000	peroxidase 1	-0.56	2.08	2.64
Os01g0327100	peroxidase 1	-0.09	2.86	2.96
Os01g0327400	peroxidase 1	-0.30	1.07	1.38
Os01g0638000	anthocyanin 3'-O-beta-glucosyltransferase	-0.04	4.00	4.05
Os01g0963000	cationic peroxidase SPC4	-0.50	1.57	2.07
Os02g0207900	caffeoylshikimate esterase	0.58	-1.75	-2.33
Os02g0240100	peroxidase 70	-0.36	1.25	1.61
Os02g0467600	cytochrome P450 CYP73A100	0.92	6.38	5.46
Os02g0697400	4-coumarate--CoA ligase 2	0.34	-0.75	-1.08
Os03g0235000	peroxidase A2	0.62	2.86	2.25
Os03g0368900	peroxidase 2	0.78	-1.35	-2.13
Os03g0406100	SPX domain-containing protein 5	-1.34	-0.10	1.24
Os04g0229100	Probable cinnamyl alcohol dehydrogenase 6	-1.16	0.05	1.21
Os04g0474800	Beta-glucosidase 12	-0.28	0.76	1.05
Os04g0651000	peroxidase 4	NA	7.84	7.89
Os04g0659300	cysteine-rich receptor-like protein kinase 6	3.82	6.69	2.86
Os04g0688300	cationic peroxidase SPC4	-0.05	1.80	1.85
Os05g0571200	probable WRKY transcription factor 41	-3.57	0.73	4.30
Os06g0547400	peroxidase P7	0.20	1.78	1.58
Os06g0549900	berberine bridge enzyme-like 18	-1.82	0.42	2.25
Os07g0601900	putative anthocyanidin reductase	1.09	4.41	3.33
Os07g0604400	COBRA-like protein 6	1.81	3.36	1.55
Os07g0677200	Peroxidase	0.03	1.07	1.05
Os07g0677500	Peroxidase	-1.78	1.09	2..87
Os08g0113000	peroxidase 47	0.31	1.61	1.30
Os08g0448000	4-coumarate--CoA ligase 5	1.30	3.19	1.89
Os08g0460000	Germin-like protein 8-14	-1.32	-0.03	1.30
Os08g0482700	blue copper protein	-0.20	0.89	1.10
Os09g0491100	Beta-glucosidase 30	-0.23	2.83	3.06
Os09g0511900	Probable inactive beta-glucosidase 33	0.34	-1.04	-1.37
Os09g0522200	Dehydration-responsive element-binding protein 1A （DREB1a）	-0.61	0.64	1.25
Os09g0544000	putrescine hydroxycinnamoyltransferase-like	1.96	6.14	4.18
Os10g0109600	peroxidase N	1.15	2.38	1.23
Os10g0379100	tryptamine hydroxycinnamoyltransferase 1-like	3.80	9.53	5.73
Os10g0380100	Tryptamine hydroxycinnamoyltransferase2	NA	6.47	6.52

Os11g0112200	Cationic peroxidase 1	-0.85	3.80	4.66
Os11g0427800	non-specific lipid-transfer protein 1-like	-0.04	3.05	3.09
Os12g0111800	cationic peroxidase 1	-0.04	5.60	5.65
Os12g0112000	peroxidase 4	0.33	2.51	2.17

Supplementary Table S7 Expression levels of genes associated with Flavonoid biosynthesis in rice seedling

**Gene_ID**	**Annotation**	**Expression level (log_2FC)**		
		**Z0 vs. Z1**	**Z0 vs. Z2**	**Z1 vs. Z2**
Os02g0467600	cytochrome P450 CYP73A100	0.07	-1.28	-1.34
Os03g0289800	probable 2-oxoglutarate-dependent dioxygenase At5g05600	-0.05	-1.20	-1.14
Os04g0581000	flavanone 3-dioxygenase 2-like	-3.52	1.15	4.68
Os04g0581100	protein DMR6-LIKE OXYGENASE 1	0.92	5.64	4.73
Os04g0611400	vacuolar-sorting receptor 7	0.34	2.03	1.69
Os05g0127500	probable 2-oxoglutarate-dependent dioxygenase At5g05600	-0.07	1.14	1.22
Os05g0526900	UDP-glycosyltransferase 88F3	-0.66	0.95	1.61
Os05g0527000	UDP-glycosyltransferase 13	-0.23	0.89	1.12
Os05g0527800	anthocyanidin 5,3-O-glucosyltransferase	-0.81	2.48	3.30
Os06g0683100	putative anthocyanidin reductase	-0.29	0.94	1.23
Os07g0510500	anthocyanidin 3-O-glucosyltransferase 2	-1.53	0.71	2.24
Os09g0544000	putrescine hydroxycinnamoyltransferase-like	2.16	7.16	4.99
Os10g0320100	flavonoid 3'-monooxygenase CYP75B3-like	-0.71	0.96	1.67
Os10g0379100	tryptamine hydroxycinnamoyltransferase 1-like	-0.05	5.52	5.57
Os10g0379854	NA	NA	5.80	5.85
Os10g0380100	NA	NA	6.47	6.52

Supplementary Table S8 Expression levels of genes associated with alpha-Linolenic acid metabolism in rice seedling

Gene_ID	Annotation	Expression level (log_2FC)		
		Z0 vs. Z1	Z0 vs. Z2	Z1 vs. Z2
Os01g0901600	NA	0.55	1.74	1.20
Os02g0194700	lipoxygenase 2.3, chloroplastic	-0.06	-1.21	-1.15
Os04g0372700	chloroplast envelope quinone oxidoreductase homolog	-0.49	0.74	1.23
Os06g0215500	Putative 12-oxophytodienoate reductase 6	NA	5.98	6.03
Os06g0215600	Putative 12-oxophytodienoate reductase 5	-1.78	1.93	3.71
Os06g0215900	Putative 12-oxophytodienoate reductase 4	-0.70	1.20	1.90
Os06g0216000	Putative 12-oxophytodienoate reductase 3	0.92	5.32	4.41
Os06g0216200	Putative 12-oxophytodienoate reductase 2	0.73	3.64	2.91
Os06g0216300	12-oxophytodienoate reductase 1	0.58	4.57	3.98
Os10g0562200	phospholipase A1-Ibeta2, chloroplastic	0.16	-1.62	-1.78
